# Prioritization and functional validation of target genes from single-cell transcriptomics studies

**DOI:** 10.1038/s42003-023-05006-7

**Published:** 2023-06-17

**Authors:** Liliana Sokol, Anne Cuypers, Anh-Co K. Truong, Ann Bouché, Katleen Brepoels, Joris Souffreau, Katerina Rohlenova, Stefan Vinckier, Luc Schoonjans, Guy Eelen, Mieke Dewerchin, Laura P.M.H. de Rooij, Peter Carmeliet

**Affiliations:** 1 Laboratory of Angiogenesis and Vascular Metabolism, Center for Cancer Biology (CCB), VIB and Department of Oncology, Leuven Cancer Institute (LKI), KU Leuven, Leuven Belgium; 2 Laboratory of Angiogenesis and Vascular Heterogeneity, Department of Biomedicine, Aarhus University, 8000 Aarhus C, Denmark; 3 Center for Biotechnology, Khalifa University of Science and Technology, Abu Dhabi, United Arab Emirates; 4 Present Address: Institute of Biotechnology of the Czech Academy of Sciences, BIOCEV, Vestec, Prague-West Czech Republic; 5 Present Address: CeMM Research Center for Molecular Medicine of the Austrian Academy of Sciences, Vienna, Austria

**Keywords:** Cell biology, RNAi, Tumour angiogenesis

## Abstract

Translation of academic results into clinical practice is a formidable unmet medical need. Single-cell RNA-sequencing (scRNA-seq) studies generate long descriptive ranks of markers with predicted biological function, but without functional validation, it remains challenging to know which markers truly exert the putative function. Given the lengthy/costly nature of validation studies, gene prioritization is required to select candidates. We address these issues by studying tip endothelial cell (EC) marker genes because of their importance for angiogenesis. Here, by tailoring Guidelines On Target Assessment for Innovative Therapeutics, we in silico prioritize previously unreported/poorly described, high-ranking tip EC markers. Notably, functional validation reveals that four of six candidates behave as tip EC genes. We even discover a tip EC function for a gene lacking in-depth functional annotation. Thus, validating prioritized genes from scRNA-seq studies offers opportunities for identifying targets to be considered for possible translation, but not all top-ranked scRNA-seq markers exert the predicted function.

## Abstract (web summary)

Harnessing existing scRNA-seq data, a set of new tip endothelial cell markers is described to demonstrate the need for targeted assessment and prioritization of candidate genes.

## Introduction

Society invests enormous resources in fundamental academic research, yet only a minor fraction (1–4%) of the achieved results is ever translated into clinical therapy^1,2^. Most academic data (even when published in top journals) are too premature and too risky for pharma to invest in. Since academics typically lack the necessary funding to provide the data required by pharma, most academic results die in the so-called valley of death^1,3^. This problem has now grown to an even larger dimension with the tsunami of single-cell/multi-omics data being generated at an increasing pace and scale every day.

Single-cell omics are revolutionizing medicine. Yet, the largely descriptive nature of single-cell RNA-sequencing (scRNA-seq) studies poses at the same time a formidable challenge—a torrent of data with long descriptive ranked lists of marker genes with a predicted putative function is produced, but (aside from validation of the transcripts at the protein level), data about the validation of the functional role of the marker genes in vitro or in vivo are scarce. It thus remains unknown which and how many of these top-ranked marker genes are also functionally relevant and, even more, might be therapeutically attractive candidates. In this exploratory study, we therefore assessed whether we could prioritize top-ranking scRNA-seq marker genes and determine how many of them were true functional targets.

Even though the human genome was sequenced nearly 20 years ago^4^, an astonishing estimated one-third of the human coding genome (nearly 6000 genes) lacks an in-depth functional annotation and/or is not or only poorly characterized in publications^5,6^—we termed them mystery genes. Demystifying the mystery genome offers enormous opportunities to obtain new biological insight and develop therapeutic strategies.

We focus on tip endothelial cells (ECs) because of several reasons. First, vessel sprouting relies on the induction of a tip EC (leading the vessel sprout)^7^, and silencing tip EC genes impairs vessel sprouting in development and disease^8^. Second, tip EC genes are the most conserved, congruent vascular markers across species, tissues, and diseases^8^. Third, specific robust methods exist to define an EC as a tip EC^9^. Fourth, one of the best-known tip EC genes is VEGFR2, the blockade of which is approved as anti-angiogenic medicine for several malignant and ocular diseases^10^. However, the success of VEGF/VEGFR2 blockade therapy is limited by resistance and insufficient efficacy^11–17^. There is thus an unmet medical need to discover novel angiogenic targets that can be considered for translation. Because of these reasons, tip ECs represent a powerful cell type to start exploring possibilities to bridge the scRNA-seq-associated valley of death.

While (tip) ECs are generally not detected at high abundance in scRNA-seq datasets, a recently published study profiled both freshly isolated and cultured tumor ECs (TECs) from human non-small-cell lung cancer (NSCLC) and peri-tumoral control lung tissue, as well as murine Lewis lung carcinoma (LLC) and control (T)ECs^8^. The combined datasets harbor >40,000 ECs (of which >3000 tip cells) and thus provide a robust in-depth view of EC heterogeneity across various models of lung cancer in different species. These datasets each contain lists of >100 top-ranking tip EC marker genes, of which only a few (*PLOD1, PLOD2, LOXL2, LXN, FSCN1*) were functionally validated in vitro (using migration and sprouting assays^8^). In vivo validation was done for *PLOD1/2* and *LOXL2* using pharmacological blockers. *LXN* and *FSCN1* were selected based on their high ranks, while *PLOD1/2* and *LOXL2* were congruent tip EC markers after meta-analyses and computational modeling^8^. Nonetheless, an exhaustive residual list of additional tip EC markers with potential translational relevance is, to date, left uncharted in terms of their functional assessment in (pathological) angiogenesis. Functional validation, however, represents one of the first yet crucial steps on the route from target identification to translation. Indeed, previous studies showed that insufficient target validation at an early stage has been linked to costly clinical failures^18,19^ and low drug approval rates^20,21^.

**Fig. 1:**
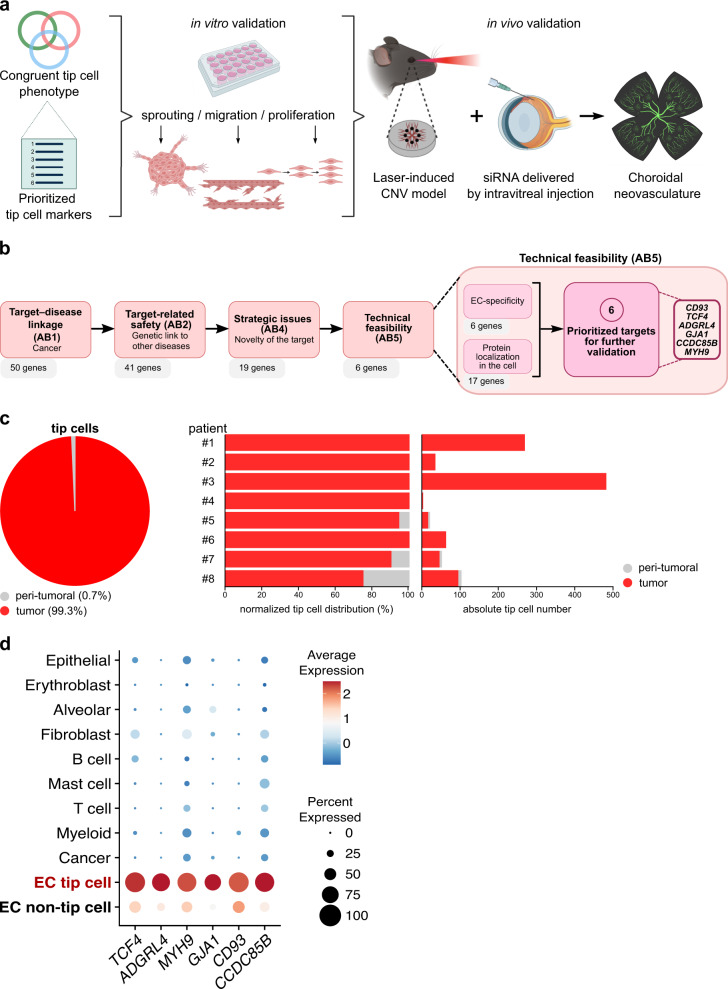
Prioritization and selection strategy. **a** Schematic representation of the study design. CNV choroidal neovascularization. Created with BioRender.com. **b** Schematic representation of the target selection strategy. AB assessment block, EC endothelial cell. **c** Left panel: pie chart representing the relative abundance of human tumor and normal (peri-tumoral) lung ECs in the tip cell subcluster. Right panel: bar plot representing relative abundances (left) and absolute cell numbers (right) of tumor and normal (peri-tumoral) lung tip cells per patient; data: Goveia et al.^8^. EC endothelial cell. **d** Dot plot heatmap of the expression of selected tip cell markers across cell types in the lung tumor microenvironment. The color intensity of each dot represents the average level of marker gene expression, while the dot size reflects the percentage of cells expressing the marker within the cell subcluster. Color scale: red—high expression, blue—low expression; data: Qian et al.^26^.

Here, by making use of EC-enriched scRNA-seq datasets across different species and disease models, we report in silico prioritization and subsequent in vitro and in vivo functional validation of previously unreported/poorly characterized tip EC marker genes (Fig. 1a).

## Results

**Fig. 2:**
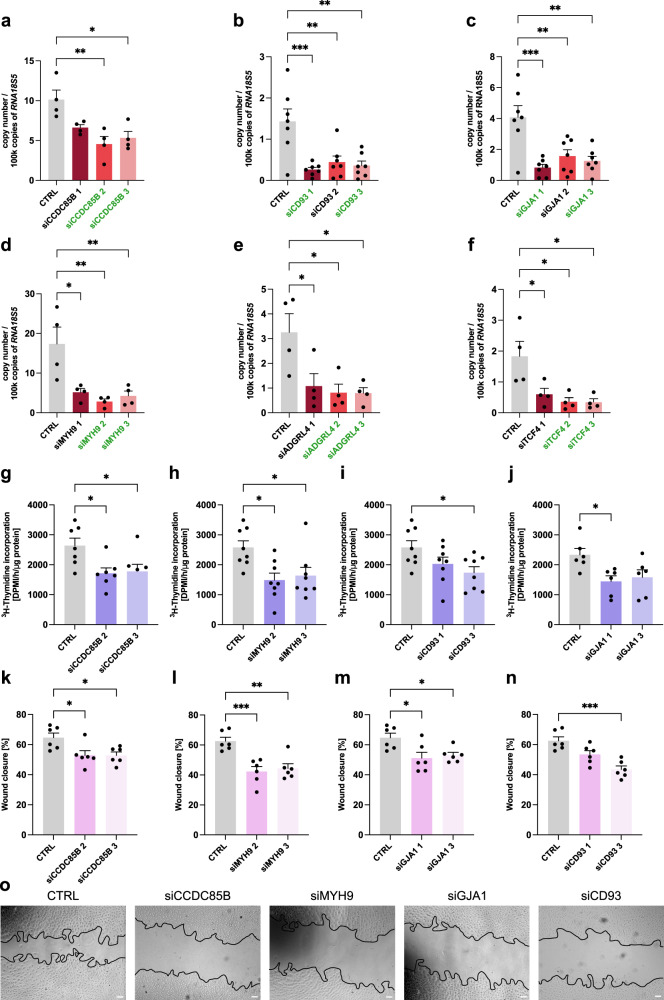
Silencing effect, proliferation & migration of tip cell markers in vitro. **a**–**f** mRNA expression levels of *CCDC85B* (**a**), *CD93* (**b**), *GJA1* (**c**), *MYH9* (**d**), *ADGRL4* (**e**), or *TCF4* (**f**) in HUVECs in control conditions and upon silencing with three independent siRNAs each, measured by RT-qPCR. Data are means ± SEM; *n* = 4 (**a**, **d**–**f**) or *n* = 7 (**b**, **c**); *p < 0.05, **p < 0.01, ***p < 0.001; by one-way ANOVA with Tukey post-hoc test for multiple group comparisons. The green font color marks the siRNA with the strongest knockdown efficiency pre-selected for further in vitro validation. Exact *p*-values: siCCDC85B: 1—0.0680, 2—0.004, 3—0.0115; siCD93: 1—0.0006, 2—0.0037, 3—0.0016; siGJA1: 1—0.0003, 2—0.0043, 3—0.0013; siMYH9: 1—0.0127, 2—0.0037, 3—0.0078; siARGRL4: 1—0.0406, 2—0.0209, 3—0.02; siTCF4: 1—0.0357, 2—0.0119, 3—0.0112. **g**–**j** Quantification of proliferation measured by [^3^H]-thymidine incorporation in control and *CCDC85B* (**g**), *MYH9* (**h**)*, CD93* (**i**), *GJA1* (**j**) silenced HUVECs. Data are means ± SEM; *n* = 7 (**g**), *n* = 8 (**h**, **i**), *n* = 6 (**j**); *p < 0.05; by one-way ANOVA with Tukey post-hoc test for multiple group comparisons. Exact *p*-values: siCCDC85B: 2—0.0243, 3—0.0363; siMYH9: 2—0.0135, 3—0.0350; siCD93 1—0.201, 3—0.0323; siGJA1 1—0.0286, 3—0.0666. **k**–**n** Quantification of migration by scratch wound assay using control and *CCDC85B* (**k**), *MYH9* (**l**), *GJA1* (**m**), or *CD93* (**n**) silenced HUVECs. Mitomycin C-treatment was used to eliminate the confounding effects of proliferation. Data are means ± SEM; *n* = 6; *p < 0.05, **p < 0.01, ***p < 0.001; by one-way ANOVA with Tukey post-hoc test for multiple group comparisons. Exact *p*-values: siCCDC85B: 2—0.0266, 3—0.026; siMYH9: 2—0.0008, 3—0.002; siGJA1: 1—0.018; 3—0.0422; siCD93 1—0.0504, 3—0.0002. **o** Representative micrographs (18 h after the scratch) showing migration in scratch wound assays using control and *CCDC85B*, *MYH9*, *GJA1*, *or CD93* silenced HUVECs. Scale bar: 50 μm. The proliferation assay (**g**–**j**) for *n* = 5 replicates was performed in the same experiment for all genes (and thus have the same control values in the quantifications); additional replicates were performed together for *CD93*, *GJA1* and MYH9 (*n* = 1) and for *CCDC85B*, *MYH9* and *CD93* (*n* = 2). The migration assay (**k**–**o**) for *n* = 5 replicates was performed in the same experiment for all genes (and thus have the same control values in the quantifications); one additional replicate of the migration assay was performed separately for *CCDC85B* and *GJA1* (donor 6) and separately for *CD93* and MYH9 (donor 7). CTRL—HUVECs transfected with control siRNA.

In order to prioritize the most promising tip cell targets from the abovementioned publicly available EC-enriched lung cancer datasets^8^, we implemented the recently published recommendations from the Guidelines On Target Assessment for Innovative Therapeutics (GOT-IT) working group^22^, which defines a framework for the process of target gene selection/prioritization (Supplementary Fig. 1a). With GOT-IT, so-called assessment blocks (ABs) are prioritized and evaluated in the context of the project and its goals (e.g., with the help of critical path questions (CPQs)). Based on AB1, 2, 4, and 5 (AB3 is not relevant for mammalian targets) and CPQs, we designed a set of prioritization criteria and applied them to the abovementioned reported tip EC marker gene list^8^, enabling prioritization of the most promising candidate targets for translation (Supplementary Data 1; Fig. 1b). Considering target–disease linkage (AB1), a focus on tip TECs was justified for several reasons. First, although the tip cell phenotype represents only a minor part of freshly isolated human (T)ECs (<10%) in lung cancer, tip cells (1) are restricted to TECs (99.3% of human tip cells originate from TECs; Fig. 1c) and (2) are the phenotype that is most sensitive to anti-VEGF treatment in the murine LLC model^8^, underscoring their relevance in pathological angiogenesis. Moreover, an integrated analysis between all three lung EC taxonomies identified a robust, congruent tip TEC marker gene signature across all species and models tested^8^. We thus continued our prioritization with the top 50 most highly ranking congruent tip TEC genes.

Based on the assessment of AB2-related concerns (target-related safety), we excluded markers with a genetic link to other diseases (related to the expression of the protein in adults). For instance, *SPARC* has been linked to disorders of the central nervous system^23^, while *SEMA6B* has been associated with progressive myoclonic epilepsy^24^. Conform AB4, we acknowledge strategic issues such as target novelty. Here, we decided to only include targets minimally described (or thus far unreported) in the context of angiogenesis and their expression in tip cells. From the top 50 congruent tip TEC signature, Goveia et al. characterized 26 of 50 genes as such (not previously described in the context of the tip cell phenotype)^8^. We updated the literature search for these remaining genes and decided to only validate markers with less than 20 publications (vaguely) describing the gene in a shared context with angiogenesis and less than three publications describing the marker to be specifically expressed in tip ECs (relevant only for *ADGRL4*, see below).

Lastly, we considered several aspects of technical feasibility (AB5), such as the availability of perturbation tools (e.g., siRNAs), protein localization in the cell (exclusion of secreted proteins)^25^, and EC specificity (Supplementary Data 1). For the latter, we analyzed the selective expression of all top 50 congruent tip cell markers in a publicly available scRNA-seq dataset of the lung tumor microenvironment (Supplementary Fig. 1b)^26^ (harboring both EC and non-EC subtypes, see “Methods”) and only included genes enriched in tip cells (versus all other lung cell types) with a log-fold change >1 (Fig. 1d; Supplementary Data 1).

The described target selection strategy resulted in six promising candidates: *CD93, TCF4, ADGRL4, GJA1, CCDC85B* and *MYH9* (Fig. 1b, d; Supplementary Data 1), which we used for further functional validation. The selected candidates are involved in various cellular functions and components, including cytoskeleton structure (*MYH9*)^27^, cell adhesion (*CD93*, *ADGRL4* (also known as *ELTD1*))^28,29^, gap junctions (*GJA1*)^30^, or regulation of gene expression (*TCF4*, transcription factor 4^31^; *CCDC85B*, putative transcriptional repressor^32^). All selected candidates are not or only minimally characterized by a function in ECs. *MYH9*, *CD93, ADGRL4, GJA1* and *TCF4* have been occasionally reported to potentially play a role in physiological and/or pathological angiogenesis^29,33–40^, *ADGRL4* has been described in only two studies as a tip cell-specific marker, yet the mechanism of function still remains unknown^41,42^, while *CCDC85B* has not been described yet (to the best of our knowledge) in the context of ECs and angiogenesis. While a few in vitro studies describe an association between CCDC85B and activation of β-catenin^43–45^, its expression pattern and potential other functions remain unknown, and it is not annotated in any of the widely used molecular pathway databases (e.g., KEGG, GO), thus far, making this mystery gene a particularly interesting candidate for follow-up investigation in relation to tip ECs and angiogenesis.

In order to assess the significance of these selected genes for classical tip EC-related functions and to explore their potential relevance for translation, we first performed siRNA knockdown (KD) experiments in vitro using primary human umbilical vein endothelial cells (HUVECs). Three different non-overlapping siRNAs were used per gene (Fig. 2a–f; Supplementary Fig. 2a–f), and the two siRNAs with the strongest KD efficiency (both at the RNA and protein level) were selected per gene for further experiments.

First, we measured the proliferative and migratory capacities of HUVECs upon siRNA-mediated KD of all six genes using ^3^H-Thymidine incorporation and wound healing assays^46^. Tip ECs are primarily involved in migration but can also proliferate, even though limitedly and contextually^47,48^. KD of *CCDC85B* reduced the proliferation of HUVECs (Fig. 2g; reduction by 35% for siRNA#2 and by 32.6% for siRNA#3, respectively). KD of *MYH9* also reduced the proliferation of HUVECs Fig. 2h; reduction by 42.3% (siRNA#2) and 36.4% (siRNA#3). A similar, however less pronounced effect (i.e., statically significant only for one siRNA construct) was observed when silencing *CD93* (32.7% reduction (siRNA#3)) and *GJA1* (38% reduction (siRNA#1)) (Fig. 2i, j). Next, we measured EC migration with wound healing assays and again observed that upon silencing of *CCDC85B*, *MYH9, CD93*, or *GJA1* HUVECs exhibited a reduction in their migration capacity (Fig. 2k–o; *CCDC85B*: reduction by 18.2% (siRNA#2, #3); *MYH9*: reduction by 32.4% (siRNA#2) and 29% (siRNA#3); *CD93*: reduction by 30.3% (siRNA#3) and *GJA1*: reduction by 21% (siRNA#1) and 18% (siRNA#3)). Silencing of the remaining genes (*ADGRL4, TCF4*) did not significantly affect EC proliferation or migration (Supplementary Fig. 2g–j).

**Fig. 3:**
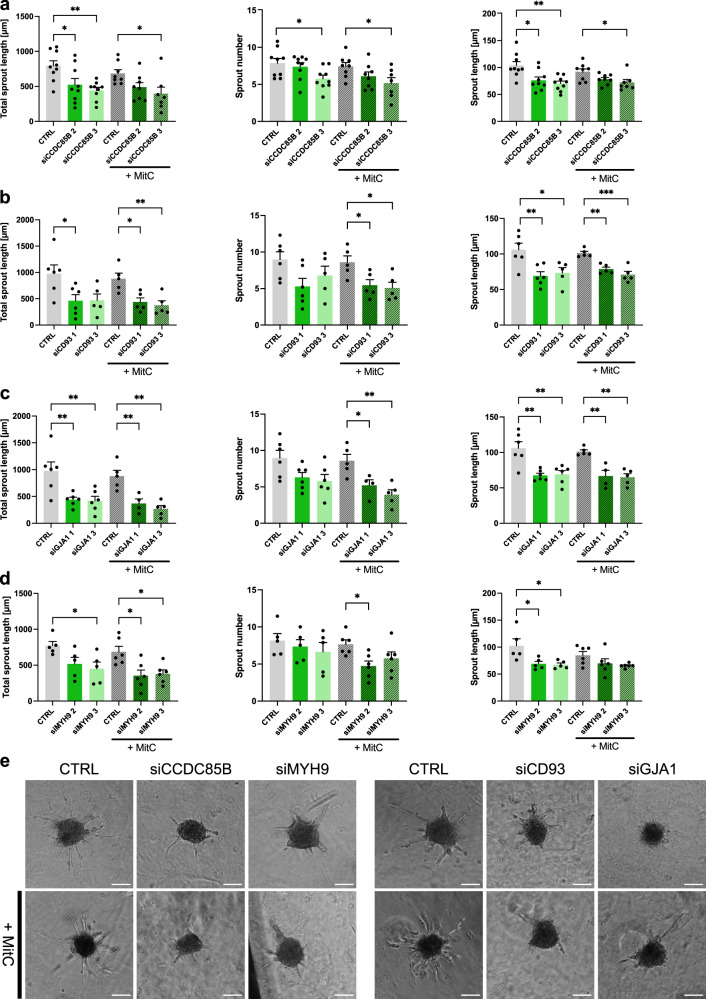
Sprouting of tip cell markers in vitro. **a**–**d** Morphometric quantification of spheroid sprouting using control and *CCDC85B* (**a**), *CD93* (**b**), *GJA1* (**c**), or *MYH9* (**d**) silenced HUVECs, with and without Mitomycin C (MitC). For all genes, three sprouting parameters were quantified: total (cumulative) sprout length, sprout number, and average sprout length. Data are mean ± SEM; *n* = 9 (**a**), *n* = 6 (**b**, **c**), or *n* = 5 (**d**); *p < 0.05, **p < 0.01; ***p < 0.001; by one-way ANOVA with the Tukey post-hoc test for multiple group comparisons. Exact *p*-values: **a** siCCDC85B: total sprout length: 2—0.0327, 2 (+MitC)—0.1518, 3—0.0048, 3 (+MitC)—0.0292; sprout number: 2—0.8137, 3—0.0358, 2 (+MitC)—0. 2874, 3 (+MitC)—0.0493; sprout length: 2—0.0119, 3—0.0022, 2 (+MitC)—0.1464, 3 (+MitC)—0.0324. **b** siCD93: total sprout length: 1—0.0422, 3—0.0561, 1 (+MitC)—0.0145, 3 (+MitC)—0.0062; sprout number: 1—0.0809, 3—0.3989, 1 (+MitC)—0.0475, 3 (+MitC)—0.02696; sprout length: 1—0.0098, 3—0.0276, 1 (+MitC)—0.0021, 3 (+MitC)—0.0002. **c** siGJA1: total sprout length: 1—0.0095, 3—0.0077, 1 (+MitC)—0.0057, 3 (+MitC)—0.001; sprout number: 1—0.1188, 3—0.0591, 1 (+MitC)—0.0337, 3 (+MitC)—0.0033; sprout length: 1—0.0019, 3—0.0027, 1 (+MitC)—0.0024, 3 (+MitC)—0.0011. **d** siMYH9: total sprout length: 2—0.111, 3—0.0406, 2 (+MitC)—0.0113, 3 (+MitC)—0.0187; sprout number: 2—0.8542, 3—0.5774, 2 (+MitC)—0.0286, 3 (+MitC)—0.184; sprout length: 2—0.0298, 3—0.0224, 2 (+MitC)—0.2546, 3 (+MitC)—0.1169. **e** Representative micrographs of spheroid sprouting assays using (left) control and *CCDC85B*, *MYH9*, or (right) control and *CD93*, *GJA1* silenced HUVECs, with (bottom) and without Mitomycin C (MitC) (top). Scale bar: 100 μm. Sprouting assays for *CD93* and *GJA1* were performed in the same experiment (*n* = 6) together with *n* = 4 replicates for *CCDC85B* and one replicate for *MYH9* (and thus have the same control values in the quantifications); two replicates of the sprouting assays for *CCDC85B* and *MYH9* were performed in the same experiment (and thus have the same control values in the quantifications), remaining replicates were assayed separately. CTRL—HUVECs transfected with control siRNA.

Using the EC spheroid sprouting assay^49^, we observed that (total) sprout length was reduced upon silencing of the same genes, for which silencing also affected EC migration in the monolayer in vitro assays (Fig. 3a–e; *CCDC85B*: reduction by 33.9% (siRNA#2) and 44.3% (siRNA#3); *CD93*: reduction by 52.6% (siRNA#1) and 52% (siRNA#3); *GJA1*: reduction by 55.3% (siRNA#1) and 57% (siRNA#3); *MYH9*: reduction by 41.6% (siRNA#3)). Since EC proliferation can influence the results of this assay, we also treated ECs with mitomycin C to block EC proliferation. When proliferation was blocked, the effect on total sprout length was similar, but also sprout numbers were significantly reduced (within the mitomycin C-treated condition) Fig. 3a–d, middle panel; reduction in sprout numbers for *CCDC85B*: by 30.5% (siRNA#3); *CD93*: by 36.6% (siRNA#1) and 40.9% (siRNA#3); *GJA1*: by 39.3% (siRNA#1) and 54.3% (siRNA#3), and *MYH9*: by 38.2% (siRNA#2). The two remaining tip cell markers did not have such a pronounced effect on EC sprouting. Silencing of *ADGRL4* showed only a small reduction (insignificant) in sprout number and sprout length alone; however, the combined effect (represented as a total cumulative sprout length) resulted in a significant reduction of HUVECs sprouting capabilities (Supplementary Fig. 3a–c; reduction by 45.6% (siRNA#2), 40.1% (siRNA#2 +MitC) and 49.2% (siRNA#3)). Silencing of TCF4 did not influence the sprouting capabilities of HUVECs (Supplementary Fig. 3d–f).

**Fig. 4:**
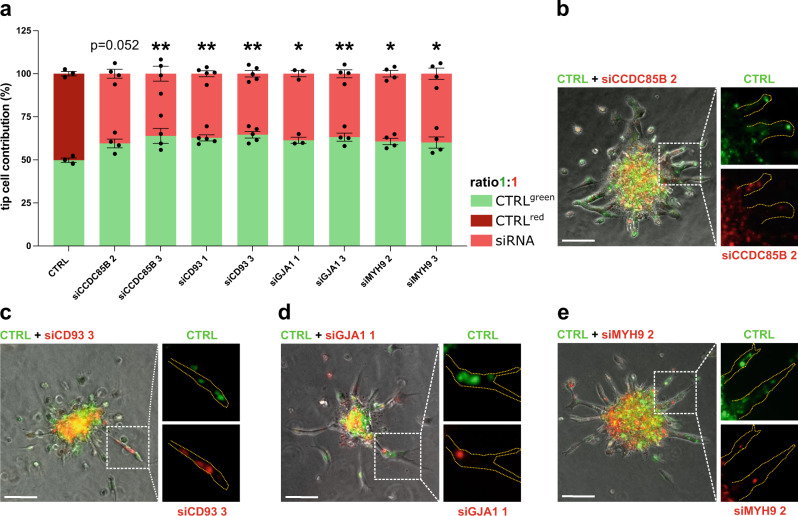
Tip cell competition in vitro. **a** The quantification of the fraction of tip cells with the indicated genotype in the tip cell competition assay using mosaic EC spheroids containing a 1:1 mixture of CTRL^green^ and CTRL^red^ (dark red) or *CCDC85B*, *CD93*, *GJA1*, or *MYH9* silenced (light red) HUVECs. Data are means ± SEM; *n* = 3 (CTRL, siGJA1 1), *n* = 4 (siCCDC85B, siGJA1 3, siMYH9), or *n* = 5 (siCD93); *p < 0.05, **p < 0.01; by χ^2^ test. Exact *p*-values: siCCDC85B: 2—0.05252, 3—0.00479; siCD93 1—0.00942, 3—0.00313; siGJA1 1—0.02102, 3—0.00756; siMYH9 2—0.03008, 3—0.04095. **b**–**e** For each panel, representative merged fluorescence micrographs of mosaic EC spheroids used for tip cell competition quantification in (**a**) are shown, as well as micrographs showing single channel images of the boxed area in the merged image. Scale bar: 100 μm. CTRL—HUVECs transfected with control siRNA.

To further assess the functional role of our most promising candidates in EC sprouting, we next examined if their silencing affected tip cell competitiveness^46^. We generated mosaic spheroids containing a 1:1 mixture of control wild-type HUVECs (green) and HUVECs silenced for tip cell markers (red) (Fig. 4a–e; Supplementary Fig. 4a). Cells with silencing of, respectively, *CCDC85B*, *CD93*, *GJA1* or *MYH9* were less often detected at the tip position (expected cell percentage on the tip cell position is 50% for control, the observed average percentages upon silencing were for *CCDC85B* 40.5% (siRNA#2) and 36.1% (siRNA#3); for *CD93* 37.2% (siRNA#1) and 35.4% (siRNA#3); for *GJA1* 38.6% (siRNA#1) and 36.8% (siRNA#3); for *MYH9* 39.3% (siRNA#2) and 40% (siRNA#3), confirming a potential tip cell-specific role of these genes (Fig. 4a–e).

To exclude the possibility that randomly selected genes from the remaining list of novel tip TEC markers would elicit similar effects on in vitro angiogenesis upon silencing, we selected *SOX4*, *SMAD1* and *FAM43A*, which, respectively, are the 24^th^, 25^th^ and 37^th^ ranking tip TEC markers, and were excluded at different stages in our target selection process (*SMAD1*—already described to be expressed in ECs and tip cells in several publications^50–52^; *SOX4*—not tip-EC specific^26^ (Supplementary Data 1; Supplementary Fig. 1b); *FAM43A* low tip-EC specificity^26^; Supplementary Data 1; Supplementary Fig. 1b). We performed a preliminary functional screen by siRNA-mediated silencing of these three targets in HUVECs (Supplementary Fig. 4b–j), and for the two siRNA constructs with the best silencing efficiency (Supplementary Fig. 4b–d), we performed functional assays. Our results show no significant effects on HUVEC migration and sprouting upon silencing each of the three candidates (Supplementary Fig. 4e–j), thus further strengthening our chosen candidate selection strategy.

**Fig. 5:**
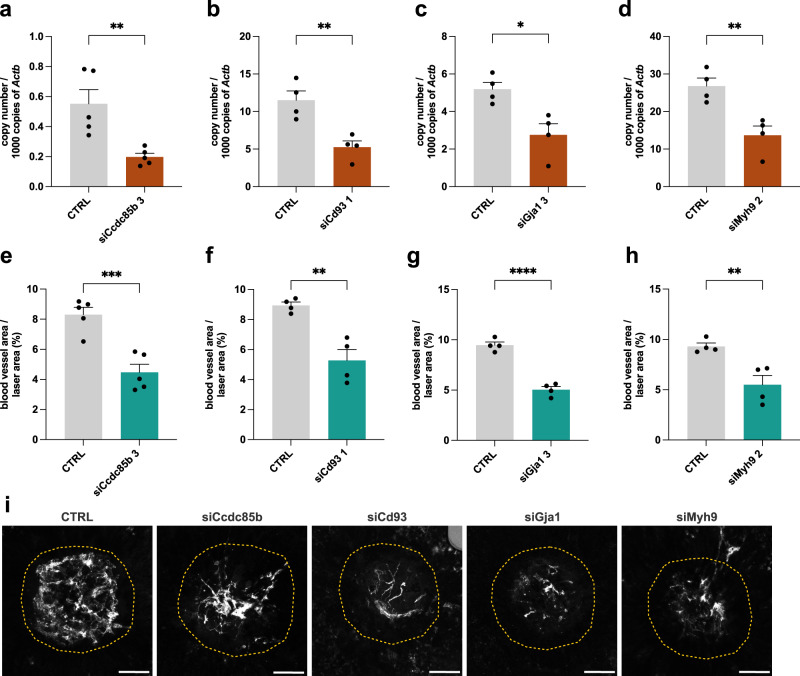
Validation of tip cell marker phenotype in vivo. **a**–**d** Expression levels of *Ccdc85b* (**a**), *Cd93* (**b**), *Gja1* (**c**), and *Myh9* (**d**), as measured by RT-qPCR in isolated murine choroidal ECs upon siRNA-mediated knockdown in vivo. Data are means ± SEM; *n* = 5 (**a**), or *n* = 4 (**b**–**d**); *p < 0.05, **p < 0.01; by unpaired two-tailed *t*-test. Exact *p*-values: siCcdc85b 3—0.0018; siCd93 1—0.0058; siGja1 3—0.0132; siMyh9 2—0.007. **e**–**h** Quantification of CNV blood vessel area in mice treated with control siRNA (CTRL) or siRNA against murine *Ccdc85b* (**e**), *Cd93* (**f**), *Gja1* (**g**), and *Myh9* (**h**). Data are means ± SEM; *n* = 5 (**e**), or *n* = 4 (**f**–**h**); **p < 0.01; ***p < 0.001; ****p < 0.0001; by unpaired two-tailed *t*-test. Each independent experiment was performed using three mice (six eyes per group). Exact *p*-values: siCcdc85b 3—0.0007; siCd93 1—0.003; siGja1 3 < 0.0001; siMyh9 2—0.0083. **i** Representative images of the neovascular area in FITC-dextran (pseudocolored white)-perfused choroidal flat mounts from mice subjected to the CNV model and treated with control siRNA (CTRL) or siRNA against murine *Ccdc85b*, *Cd93*, *Gja1*, and *Myh9*. The laser area is marked with a yellow dashed line. Scale bar: 75 μm. CTRL—cells in vivo transfected with control siRNA.

Wet age-related macular degeneration (AMD) is a leading cause of blindness in the elderly worldwide^53^. This condition is characterized by the presence of choroidal neovascularization (CNV), in which new immature blood vessels grow toward the outer retina from the underlying choroid^53^. CNV often results in dysfunctional and leaky vessels^54^, thereby resembling tumor blood vessels in terms of pathological morphology^55^. In order to investigate the effect of tip cell markers and their function on vessel sprouting in vivo, we utilized the laser-induced CNV assay—a validated mouse model used extensively in a wide range of studies focused on angiogenesis^56^, in combination with intravitreal injection of siRNAs targeting respectively *Ccdc85b, Cd93, Gja1*, *Myh9, Adgrl4* or *Tcf4*. While siRNA-mediated targeting is not EC-specific in this experimental setup, we confirmed that our candidate genes were consistently enriched in the EC-compartment of human and mouse eye transcriptomics datasets (Supplementary Fig. 5a). We screened three different siRNAs using murine brain microvascular endothelial cells (bEnd3) and pre-selected the siRNA giving the strongest silencing (Supplementary Fig. 5b–g). Next, we performed laser-induced lesioning of the choroid in mice (day 1), followed by intravitreal injection of selected siRNAs on days 1 and 4. On day 7, we sacrificed the mice after FITC-dextran perfusion and harvested the choroids both for KD assessment (Fig. 5a–d; Supplementary Fig. 5h, i) and histological analysis (Fig. 5e–i; Supplementary Fig. 5j, k). To confirm the silencing at the transcriptomic level, we isolated a highly pure EC population by digestion of choroidal tissue and FACS sorting (Supplementary Fig. 6a)^57^. The choroids for histological analysis (with vasculature stained by FITC-dextran perfusion) were flat-mounted. Morphometric measurement of the area of neovascularization showed that silencing of *Ccdc85b*, *Cd93, Gja1* or *Myh9* resulted in a significant reduction of neovascularization in laser-induced CNV (46.2% reduction upon silencing of *Ccdc85b*, 46.8% for *Gja1* and 41% reduction upon silencing of both *Cd93* and *Myh9*; Fig. 5e–i), confirming their involvement in sprouting angiogenesis in vivo. Moreover, we observed that silencing of *Adgrl4* resulted in a moderate reduction (27.3%, however not reaching statistical significance) of the neovascularized area (Supplementary Fig. 5j, k), whereas silencing of *Tcf4* did not have any impact on choroidal neovascularization (Supplementary Fig. 5j, k), in line with the lack of any effect on tip cell function/angiogenesis observed in our in vitro experiments. Altogether, these results strongly confirm the value and robustness of our gene prioritization strategy.

## Discussion

In this study, we took advantage of EC-enriched, publicly available scRNA-seq studies that created a robust list of conserved tip cell markers congruent across diseases, models, and species^8^. Typically, cell phenotypes are clustered based on the expression of 50 to 100 top-ranking markers, making it highly challenging / not feasible for regular academic laboratories to functionally validate all putative targets. In addition, the highest top-ranking markers are not necessarily functionally the most relevant or therapeutically the most attractive. To address this shortcoming, we created a universal selection strategy using GOT-IT target assessment recommendations^22^ and prioritized six promising targets (*CCDC85B*, *CD93*, *GJA1*, *MYH9*, *ADGRL4*, *TCF4*). Functional validation confirmed the success of our selection strategy and revealed that in vitro silencing of four of these potentially novel tip cell markers resulted in the impairment of EC function and impaired EC sprouting. Moreover, silencing of these four targets in vivo in a model of pathological sprouting angiogenesis (CNV) reduced the neovascular area. Altogether, our results confirm the predictive value of single-cell studies, highlight the importance of functional validation of tip EC markers and provide an attractive and valuable approach for using single-cell datasets to prioritize candidates for translation. Importantly, and pending available models for functional validation, our selection strategy is very likely expandable to other cell types and conditions and may thus have a broad range of implications. Our target prioritization strategy moreover resulted in the selection of *CCDC85B*—a gene with minimally described functions to date. Such ‘mystery genes’, awaiting detailed functional annotation, make up nearly a third of the human coding genes and thus represent a formidable unmined opportunity to gain knowledge, develop new therapeutic strategies, and bridge the valley of death.

It is worth mentioning that we performed in vivo validations on all six in vitro tested candidates since the negative outcome of our functional validation in vitro for *TCF4* and *ADGRL4* does not necessarily disqualify them as putative tip EC markers. Indeed, our current knowledge about tip ECs may still be incomplete, and perhaps, *TCF4* and *ADGRL4* are involved in yet unrecognized tip EC functions. In fact, we observed a significant reduction in total sprout length in vitro (Supplementary Fig. 3a) and a small (but statistically insignificant) reduction of choroidal neovascularization (Supplementary Fig. 5j, k) upon silencing of *Adgrl4* in vivo, in line with a handful of studies showing the involvement of ADGRL4 in sprouting angiogenesis^29,36,41,42,58^. Yet, our in vitro and in vivo results with other candidate genes were more robust. The precise function of TCF4, except its involvement in neural development^59^, remains mostly unknown. Silencing of *Tcf4* in vivo did not significantly affect neovessel formation, in line with the lack of any effect on tip cell function / angiogenesis in our in vitro experiments. Although preliminary, our results suggest that in vitro assessments of EC angiogenic function may serve as a good proxy for the in vivo angiogenic role of a given gene or protein. Moreover, these findings further stress the importance of functional validation of in silico identified targets and, importantly, also the need for (ideally even more) precise predictive selection strategies.

We acknowledge that our study also faces limitations. First, our pre-selected candidate genes are limited to the top 50 most highly ranking congruent tip TEC markers identified across two human and one murine lung cancer datasets^8^. For a more detailed prioritization strategy, a broader set of data (including other types of cancers/diseases and models) and/or applying more stringent parameters to define and rank the most tip EC-specific marker genes from the original datasets could be instrumental. Second, inconsistencies in the correlation between mRNA and protein expression levels could result in unexpected results during functional validation of findings derived from transcriptomics data. Moreover, as silencing of one tip cell marker may not be sufficient to affect vessel sprouting, the synergistic effect by combinatorial silencing of more than one tip cell marker should be considered. Third, the prioritization strategy is limited to aspects that can be selected and analyzed based on a manual literature search. Given the tsunami of data that is being generated by various state-of-the-art single-cell omics technologies, a more high-throughput and automated approach (e.g., text mining and artificial intelligence (AI)-based tools) may be needed to fully explore the potential of these data, especially in the context of target discovery for translation. Despite the exciting prospects of AI/text mining-based methods for future gene prioritization, we are well aware of the fact that this may not be an accessible approach for research groups with minimal computational expertise and/or resources. For these, we envision that a manual literature search would still be a valuable option, especially when used in combination with a more thorough meta-analysis of a larger set of (single-cell) datasets to obtain a list of ranked candidate genes. Candidate genes that rank highly in a specific condition or cell type across multiple studies have a higher likelihood of being biologically relevant markers/targets. Even though a manual literature search, in this case, will still be required to unravel the novelty of the candidates, the overall concerted effort of gene prioritization will likely become the preferred method for target selection. Even when (computational) resources are limited, recent developments in the area of user-friendly, free-of-charge cloud-based data analytic tools (e.g., Cellenics, https://www.biomage.net/) should allow researchers of different backgrounds to comprehensively mine multiple omics datasets. Fourth, in vitro systems often do not allow to assess the function of a specific subcluster, and gene silencing in the CNV model is not EC-specific, stressing the urgent need for selective, EC-subtype specific in vivo validation models. Fifth, the translational implications of our study need to be further investigated in additional pre-clinical models (e.g., tumor models, etc.), including aspects such as drug/target design and delivery, perturbation efficiency, and toxicity. Finally, more in vitro and in vivo studies have to be performed to investigate and describe mechanisms beyond the effect of selected genes on tip cell function and phenotype. Notwithstanding these shortcomings, our study provides an assessment of the functional contribution of in silico prioritized tip cell marker genes to in vitro angiogenesis, as well as to pathological angiogenesis (CNV) in vivo. Our study highlights both the significance and potential of detailed (descriptive) single-cell studies, as well as the importance of target selection, wet-lab assessment of bioinformatically discovered targets, and demonstrates the potential translational relevance of *CCDC85B*, *CD93, MYH9*, and *GJA1*. Finally, our strategy promises to discover the function of mystery genes as a first step toward demystifying the mystery genome.

## Methods

### List of congruent tip TEC markers

The list of congruent tip TEC markers was extracted from a publicly available lung tumor study^8^. In brief, to identify congruent tip cell markers across freshly isolated human and murine TECs, as well as cultured human TECs, all genes that were most highly expressed in tip cells and breach cells (only in murine data—an ECs subpopulation with similar transcriptomic pattern to the tip cells^8^) across these three datasets were selected. Second, these conserved genes were ranked via a rank-product meta-analysis by calculating the product of the rank numbers of each gene in each of the three datasets^60,61^, and (Benjamini–Hochberg) correction of the associated *p*-values using the R package q value^62^.

### Target selection and prioritization of tip cell markers

To select the most promising targets for functional validation, we used the list of the top 50 most congruent tip cell marker genes^8^ and followed recommendations described by the GOT-IT (Guidelines On Target Assessment for Innovative Therapeutics) working group^22^. In detail, we designed a set of selection categories relevant to our experiments based on GOT-IT Assessment Blocks (Supplementary Fig. 1a) and critical path questions (CPQs)^22^ (Supplementary Data 1).

1. AB1—target–disease linkage: an assessment of whether genes harbor a relevant link to disease (cancer/pathological angiogenesis) was made. We performed the analysis of tip cell cluster distribution across tumor and normal (peri-tumoral) samples (using the human lung single-cell transcriptomic data^8^) with BIOMEX^63^. Tip TECs are almost exclusively found in tumor-derived samples in both mouse and human lung cancer—99.3% of human tip cells were derived from tumor samples (Fig. 1c). Conform this assessment, the meta-analysis based on Goveia et al. allowed us to include all top 50 ranking tip TEC markers in our analysis^8^.
2. AB2—target-related safety: we screened the literature for genes with a direct genetic link to other diseases (see details in Supplementary Data 1) and excluded genes with expression levels linked to the development of a disorder in adults.
3. AB3—is related to microbial targets and was therefore not included in the pre-selection process.
4. AB4—strategic issues: we considered target gene novelty and assessed whether a gene was not previously described to be expressed in tip cells. We updated the literature search for the 26 genes reported as a novel by Goveia et al.^8^ and decided to validate only never or minimally characterized tip cell markers, i.e., less than 20 publications (vaguely) describing the gene in a shared context with angiogenesis and less than three publications describing the marker to be specifically expressed in tip ECs (relevant only for *ADGRL4*).
5. AB5—technical feasibility: first, we confirmed the assessability in in vitro and in vivo angiogenic assays—for all genes, pre-validated siRNA and shRNA against both murine and human targets were commercially available. Next, to directly target ECs, we excluded target genes whose product is secreted, as secreted factors can affect many other cells (Open Target Platform^25^; GeneCards—highest confidence score of protein localization).

Lastly, we evaluated the EC specificity of the tip cell markers based on a recently published lung cancer atlas, in which the entire tumor microenvironment was profiled (including ECs and non-EC cell types). Briefly, lung data and accompanying metadata was downloaded from: https://lambrechtslab.sites.vib.be/en/pan-cancer-blueprint-tumour-microenvironment-0^26^. The EC subset of the processed data was separately subclustered and annotated using previously reported EC-subtype specific marker genes^8,64^. Briefly, endothelial cells were selected based on the annotations provided by the study^26^ (*subset* function of Seurat (v3.1.5)^65^, idents = ‘EC’). Data were normalized using the *NormalizeData* function, followed by the identification of the top 2000 highly variable genes using *FindVariableFeatures*, and scaling of the data using the *ScaleData* function. The scaled data was then summarized by principal component analysis (PCA; *RunPCA* function) and subclustered (*FindClusters* function, Resolution=1), followed by visualization using uniform manifold approximation and projection (UMAP; *runUMAP* function). EC clusters were annotated based on the expression of known EC and non-EC marker genes, including *GJA5* and *CXCL12* (arterial ECs), *EDNRB, HPGD, TMEM100, BTNL9* (microvascular ECs), *ACKR1* and *VCAM1* (venous ECs) *PROX1* and *LYVE1* (lymphatic ECs), *APLN* and *PGF* (tip ECs), *PTPRC* and *CD68* (immune cells), *DCN, LUM, PDGFRB* (stromal cells), *EPCAM, CDH1* (epithelial cells). Contaminating immune cell clusters, as well as clusters without any clear EC marker gene expression (but high expression of ribosomal genes and/or a relatively lower number of detected genes), were removed, and all downstream analysis was performed on the finally selected ECs only (*n* = 3448 cells). EC subclusters were annotated as either tip or non-tip EC, and the ECs were combined again with all non-EC cell types profiled in this dataset (cancer, alveolar, epithelial, myeloid, T, B and mast cells, fibroblasts and erythroblasts). To determine the specificity of our candidate markers to the tip EC cluster, we calculated marker genes for every cell type using the *FindAllMarkers* function (only.pos=TRUE, max.cells=1000, all other parameters were default) (Supplementary Fig. 1b). Tip cell markers enriched in the tip EC cluster with a log-fold change >1 (as compared to all other cell types) were considered tip cell and EC specific. After applying all the abovementioned criteria, six potential target genes emerged: *CCDC85B*, *CD93*, *GJA1*, *ADGRL4* and *TCF4* (Fig. 1d).

### Cell lines and primary cell culture

#### Human umbilical vein endothelial cells

##### Ethical approval

Human umbilical vein endothelial cells (HUVECs) were freshly isolated from umbilical cords obtained (soon after birth) from multiple donors of unknown sex with approval from the Ethics Committee Research KU Leuven/UZ Leuven (approval number S57123). Informed consent was obtained from all subjects (parents).

##### HUVEC isolation

Human umbilical vein endothelial cells (HUVECs) were freshly isolated from umbilical cords^66^. Briefly, the interior of the umbilical vein was rinsed with PBS containing antibiotic-antimycotic solution (Thermo Fisher Scientific) and injected with pre-heated collagenase I solution (0.2% collagenase type I in 0.9% NaCl, 2 mM CaCl_2_, antibiotic-antimycotic). After no more than 13 min incubation, the collagenase suspension containing endothelial cells (ECs) was collected, filtered through a 40-μm nylon cell strainer, and spun down. The ECs were plated on 0.1% gelatin-coated dishes in M199 medium (1 mg/mL D-glucose) (Thermo Fisher Scientific) supplemented with 20% fetal bovine serum (FBS) (Merck-Biochrom), 2 mM L-glutamine (Thermo Fisher Scientific), Endothelial Cell Growth Supplement (ECGS)/ Heparin (PromoCell), 100 IU/mL penicillin and 100 μg/mL streptomycin (Thermo Fisher Scientific), and cultured until confluent in a 5% CO_2_, 37 °C incubator. The confluent cultures were split and replated in a 1:1 mixture of M199 and endothelial cell basal medium (EGM2) (PromoCell) supplemented with endothelial cell growth medium supplement pack (PromoCell) and further cultured in EGM2 medium. In all experiments, HUVECs were used as single-donor (biological repeats) cultures (explaining occasional variability in some assays due to slight inter-donor variability of the HUVEC responses) and were used between passages 2 and 5. Cultures were regularly tested for mycoplasma.

### bEnd3 murine endothelial cells

Commercially available bEnd3 cells (murine, brain endothelial cells; ATCC, France) were cultured in high glucose DMEM supplemented with 10% FBS (fetal bovine serum), 2 mM L-glutamine, and 1% penicillin/streptomycin (see details in Supplementary Table 1).

### Mice

Experiments were performed in 7- to 10-week-old C57BL6/J mice obtained from the KU Leuven animal facility. Animals were maintained in individually ventilated cages in a room with controlled temperature (22 ± 2 °C) and humidity, under a 12-h light/12-h dark cycle, and with food (ssniff R/M-H diet, V153x) and drink ad libitum. Animals were closely followed-up by the animal caretakers and the experimenters, with regular inspection by a veterinarian, as per the standard health and animal welfare procedures of the local animal facility. No statistical method was used to predetermine the sample size. Animal housing and all experimental procedures were approved by the Institutional Animal Ethics Committee of the KU Leuven (Belgium) under protocol number P077/2021.

### Mouse model of choroidal neovascularization

Choroidal neovascularization (CNV) was induced by laser burns using a Purepoint Laser (Alcon, Fort Worth, USA)^67^. Ten or eight impacts (for choroidal endothelial cell isolation or histological analysis, respectively) rupturing the Bruch’s membrane were made around the optical nerve using a laser diameter of 100 μm, power 0.320 W, and exposure time of 0.05 s in both eyes. On day 7, at the height of the angiogenic response^56^, mice were euthanized by cervical dislocation (three mice (six eyes) per condition), and the eyes (for histological analysis) were enucleated 10 min after retrobulbar injection with Fluorescein isothiocyanate (FITC)-conjugated dextran (Mr 2,000,000) (Sigma-Aldrich) and fixed in 2% paraformaldehyde. Choroids were dissected, flat-mounted (ProLong Gold antifade reagent, Thermo Fisher Scientific), and imaged using a Leica TCS SPE confocal microscope (Leica Microsystems). Analysis of the neovascular area was performed with the Leica MM AF morphometric analysis software (Leica Microsystems) and expressed as the FITC-dextran positive area in percent of the total CNV lesion area. This procedure was repeated for 4–5 independent replicate experiments, each: three mice (six eyes) per condition.

### Murine choroid EC Isolation

Choroidal endothelial cells were isolated based on the protocol published by Conchinha et al.^57^, omitting the magnetic cell sorting steps. In brief, on day 7 after the laser-induced CNV (as described above), the mice were sacrificed by cervical dislocation, and eyes were collected by inserting scissors along the eye into the orbital cavity. The four optical muscles and the optic nerve, which appear as a white cord behind the eye, were cut. The dissected eyes were then placed in PBS and periocular tissue was removed. The retinal pigment epithelium (RPE)-choroid-sclera complex was dissected from the enucleated eyes by peeling off the vitreous body and retina. The choroids were dissociated into single-cell suspension in a digestion buffer (0.3% (w/v) collagenase I, DNase I (7.5 μg/mL) and dispase (0.25 U/mL) in Knock-Out^TM^ DMEM-medium (Thermo Fisher Scientific) supplemented with 1 mM sodium pyruvate, 1x MEM NEAAs, ECGF/Heparin, antibiotic/antimycotic (2x) and 1% (v/v) penicillin/streptomycin) for 30–40 min at 37 °C with manual pipetting every 10 min. The reaction was stopped with 5 mL of wash buffer^57^, and the cell suspension was filtered through a 100- and 40-μm cell strainer. The choroidal endothelial cells were sorted using fluorescence cytometry^57^. Single-cell suspensions were stained with viability dye (VD, eFluor™ 450, dilution 1:1000) and fluorescently labeled antibodies (CD45 (PE-Cy7, dilution 1:500), CD31 (AF488, dilution 1:100) and CD102/ICAM2 (APC, dilution 1:50)) for 30 min. Then, we FACS-sorted viable single cells (VD^−^), CD45^−^, CD102^+^ and CD31^+^ directly into the lysis buffer from the RNeasy Micro Kit (Qiagen). We based the selection of ECs both on CD31 and CD102 to increase purity (Supplementary Fig. 6a). Sorted choroidal EC were subjected to RNA isolation (RNeasy Micro Kit (QIAGEN); SuperScript III First Strand cDNA synthesis kit (Thermo Fisher Scientific)) and quantitative RT-PCR. This procedure was repeated for 4–5 independent replicate experiments, each: three mice (six eyes) per condition.

### In vitro functional assays

#### Proliferation

EC proliferation was quantified by incubating cells for 2 h with 1 μCi/mL [^3^H]-thymidine (Perkin Elmer). Thereafter, cells were fixed with 100% ethanol for 15 min at 4 °C, precipitated with 10% TCA and lysed with 0.1 M NaOH. The amount of [^3^H]-thymidine incorporated into DNA was measured by scintillation counting.

### Scratch wound assay

A scratch wound was applied on confluent EC monolayers (pre-treated with 4 μg/mL Mitomycin C for 6 h) using a 200 μL tip; 24 h after seeding (100,000 cells per well in 24-well plates). After scratch wounding and imaging at time point 0 (T0), the cultures were further incubated in a fully supplemented EGM2 medium for 18 h and imaged again (T18). Migration was measured using the Fiji/ImageJ software package and is expressed as % wound closure (gap area at T0 minus gap area at T18 in % of gap area at T0).

### Spheroid capillary sprouting assay

ECs were incubated overnight in hanging drops in EGM2 medium containing methylcellulose (20% (v/v) of a 1.2% solution of methylcellulose 4000 cP (Sigma-Aldrich)) to form spheroids. When mitotic inactivation was required, Mitomycin C (2 μg/mL) was added to this medium. Spheroids were then embedded into a collagen gel and cultured for 24 h to induce sprouting^46^. Cultures were fixed with 4% paraformaldehyde (PFA) at room temperature (RT) and imaged (bright field) with a Leica DMI6000 microscope (Leica Microsystems); at least 10 spheroids were imaged per condition. Analysis of the number of sprouts per spheroid, individual sprout length, and the total sprout length (cumulative length of primary sprouts and branches per spheroid) was performed on phase-contrast images using the Fiji/ImageJ analysis software.

### Mosaic spheroid capillary sprouting assay (tip cell competition assay)

Control and silenced ECs were generated as described in the knockdown strategy section (see below) and fluorescently labeled with intracellular dyes. Control and silenced ECs were stained respectively with the CYTO-ID® Green and CYTO-ID® Red long-term cell tracer kit (Enzo) according to the manufacturer’s guidelines (Supplementary Fig. 4a). Briefly, suspensions containing control or silenced HUVECs were placed in a separate tube, washed, and spun down twice with 1X HBSS. Then cells were incubated in a labeling solution with the dye for 7 min at RT in the dark according to the manufacturer’s instructions. The staining reaction was stopped with the stop buffer, and cells were washed with an excess of full EGM2 medium to remove the remaining free dye in the solution, the cells were centrifuged, and the supernatant was removed. Finally, control (Green) and silenced (Red) cells were counted, mixed at an equal ratio (1:1, 250,000 cells in total), and used for spheroid formation and sprouting assays as described above. Using a Leica DMI6000 fluorescence microscope (Leica Microsystems), at least 15 spheroids were imaged and quantified per condition. Using the Fiji/ImageJ analysis software package, the percentages of sprouts with either a green-stained or a red-stained EC occupying the tip position were calculated. Please note that the appearance of labeled cells may vary slightly from uniformly bright to punctuate. This difference is caused by the extent of membrane internalization occurring from the moment of cell labeling throughout the total length of the mosaic spheroid assay (68–72 h) (Supplementary Fig. 4a).

### Knockdown strategy

#### In vitro

HUVECs: To silence the expression of *GJA1*, *CD93*, *CCDC85B*, *MYH9*, *TCF4*, and *ADGRL4* (*ELTD1*) in the in vitro cell culture model using (HUVEC) primary cells, siRNA duplexes directed against the human genes mentioned above and control DsiRNA (NC1) (IDT Integrated DNA Technologies) were used. To induce the silencing, HUVECs were transfected using the lipofectamine RNAiMAX transfection kit (Thermo Fisher Scientific) as described by the manufacturer with the following modifications: the volume of Lipofectamine RNAiMAX Reagent was reduced by 30%, and the concentration of DsiRNA was increased to 20 μM (instead of the recommended 10 μM) in order to obtain efficient knockdown with limited toxicity on the HUVECs. Twenty hours after transfection with DsiRNA-lipid complexes, the cells were washed and cultured for at least 24 h before use in the in vitro functional assays. For quantification of the silencing effect by Western blotting and quantitative RT-PCR, the cells were cultured for 48–90 h after transfection.

bEnd3: To silence the expression of *Gja1*, *Cd93*, *Ccdc8bB, Myh9, Adgrl4, or Tcf4* in bEnd3 murine cells, duplexes directed against the murine genes mentioned above and control DsiRNA (NC1) (IDT Integrated DNA Technologies) were used. bEnd3 cells were transfected using the Lipofectamine RNAiMAX transfection kit (Thermo Fisher Scientific) as described above. Twenty-four hours after transfection with DsiRNA-lipid complexes, the cells were washed and cultured for 24 h before harvesting for quantification of the silencing effect by quantitative RT-PCR.

#### In vivo

To silence the target genes in the CNV model in vivo, pre-selected siRNA duplexes directed against murine *Ccdc85b*, *Cd93*, *Gja1*, *Myh9, Adgrl4, Tcf4* or control DsiRNA (NC1) (IDT Integrated DNA Technologies) were intravitreally injected (1 μg/eye; injection volume: 1 μL, with the in vivo-jetPEI® reagent according to the manufacturer’s instruction) immediately after induction of the laser burns and re-injected (to sustain the transient effect of siRNA treatment^68^) at day 4 after induction of the laser burns with the same DsiRNA concentration (1 μg/eye)^67,69^. On day 7 post-induction, the eyes were enucleated and processed for analysis of the neovascular area as described above. In order to check the genetic silencing in ECs upon DsiRNA treatment, on day 7 post-induction, choroidal ECs were isolated, single-cell suspensions were generated as described above, and alive, single-cell, CD45^−^, CD31^+^, CD102^+^ choroidal ECs were FACS sorted (BD FACSAria III) (Supplementary Fig. 6a). The sorted EC cells were then immediately processed for RNA extraction and qRT-PCR was performed.

### RNA isolation and quantitative RT-PCR

RNA was collected and purified with the PureLink RNA Mini Kit (Thermo Fisher Scientific; for RNA isolation from HUVECs and bENd3 cells) or RNeasy Micro Kit (QIAGEN; for RNA isolation from choroidal ECs) and converted to cDNA using the iScript cDNA synthesis kit (Bio-Rad) or the SuperScript III First Strand cDNA synthesis kit (Thermo Fisher Scientific), respectively. RNA expression analysis was performed with TaqMan Fast Universal PCR Master Mix (Thermo Fisher Scientific) using premade primer sets (IDT Integrated DNA Technologies). For comparison of gene expression between conditions, mRNA levels were normalized to the housekeeping gene *RNA18S5* (human) or *Actb* (murine).

### Protein extraction and immunoblotting

Protein extraction and immunoblot analysis were performed using a modified Laemmli sample buffer (125 mM Tris-HCl, pH 6.8 buffer containing 2% SDS and 10% glycerol) or RIPA Lysis and Extraction Buffer (Thermo Fisher Scientific) in the presence of protease and phosphatase inhibitors (Roche). Lysates were separated by SDS-PAGE under reducing conditions, transferred to a nitrocellulose or PVDF membrane, and analyzed by immunoblotting. Primary antibodies and appropriate secondary antibodies are listed in Supplementary Table 1. The signal was detected using the ECL or Femto system (Thermo Fisher Scientific) according to the manufacturer’s instructions. Densitometric quantifications of bands were done with Fiji software.

### Analysis of single-cell transcriptomics datasets of the human and mouse eye

#### Human

Data from Voigt et al.^70^ were downloaded from the Gene Expression Omnibus (GEO; accession number GSE135922) and analyzed using Seurat (v3.1.5)^65^. Briefly, the data was normalized (*NormalizeData* function), followed by the identification of the top 2000 highly variable genes (*FindVariableFeatures* function) and scaling of the data (*ScaleData* function). The resulting data was then summarized by principal component analysis (PCA; *RunPCA* function) and subclustered (*FindClusters* function, resolution 0.5, using the top-25 principal components), followed by visualization using uniform manifold approximation and projection (UMAP; *runUMAP* function). Subclusters were annotated based on cellular lineages and reported marker genes in the original publication, and the *DotPlot()* function was used for dot plot heatmap visualization of marker genes. Data from Lehman et al.^71^ were downloaded from GEO (accession number GSE135167). Log-fold change values were extracted for every candidate gene, followed by heatmap visualization using the *ComplexHeatmap* R package (2.12.1; Euclidean distance, average linkage). *Tcf4* was not present in this dataset.

#### Mouse

Data from Lehman et al.^71^ were downloaded from GEO (accession number GSE135167). Log-fold change values were extracted for every candidate gene, followed by heatmap visualization using the *ComplexHeatmap* R package (2.12.1; Euclidean distance, average linkage). *Tcf4* was not present in this dataset.

### Statistics and reproducibility

In vitro and in vivo functional tests were performed using at least three independent biological repeats as specified in the respective legends. Each independent replicate of the in vivo CNV assay was performed using choroids from three mice (six eyes) per condition. Data are represented as mean ± SEM. Source data of the quantified data of the main figures are listed in Supplementary Data 2. When comparing two groups for a single parameter, an unpaired two-tailed *t*-test was used, while for three groups, one-way ANOVA with Tukey post-hoc test for multiple group comparisons tests was used (in GraphPad Prism10), a χ^2^ test was used for mosaic spheroid assay; *p*-value < 0.05 was considered significant.

### Reporting summary

Further information on research design is available in the Nature Portfolio Reporting Summary linked to this article.

## Supplementary information

Supplementary Information

Description of Additional Supplementary Files

Supplementary Data 1

Supplementary Data 2

Reporting Summary

## Data Availability

The publicly available lung cancer EC data used in this study are available in the ArrayExpress database at EMBL-EBI under accession code E-MTAB-6308 and at https://carmelietlab.sites.vib.be/en/software-tools (lung Tumor ECTax). The publicly available lung cancer data used in this study are available in the ArrayExpress database at EMBL-EBI under accession code E-MTAB-8107 and at https://lambrechtslab.sites.vib.be/en/data-access. The publicly available human and mouse eye data used in this study are available at the Gene Expression Omnibus (GEO) under accession numbers GSE135922 (human) and GSE135167 (mouse). Source data are provided with this paper. Uncropped and unedited blot images are presented in Supplementary Fig. 7.

## References

[1] Seyhan AA (2019). Lost in translation: the valley of death across preclinical and clinical divide—identification of problems and overcoming obstacles. Transl. Med. Commun..

[2] Hudson J, Khazragui HF (2013). Into the valley of death: research to innovation. Drug Discov. Today.

[3] Fernandez-Moure JS (2016). Lost in translation: the gap in scientific advancements and clinical application. Front. Bioeng. Biotechnol..

[4] Collins FS, Fink L (1995). The Human Genome Project. Alcohol Health Res. World.

[5] Wood V (2019). Hidden in plain sight: what remains to be discovered in the eukaryotic proteome?. Open Biol..

[6] Dey G, Jaimovich A, Collins SR, Seki A, Meyer T (2015). Systematic discovery of human gene function and principles of modular organization through phylogenetic profiling. Cell Rep..

[7] Potente M, Gerhardt H, Carmeliet P (2011). Basic and therapeutic aspects of angiogenesis. Cell.

[8] Goveia J (2020). An integrated gene expression landscape profiling approach to identify lung tumor endothelial cell heterogeneity and angiogenic candidates. Cancer Cell.

[9] Nowak-Sliwinska P (2018). Consensus guidelines for the use and interpretation of angiogenesis assays. Angiogenesis.

[10] Carmeliet P, Jain RK (2011). Molecular mechanisms and clinical applications of angiogenesis. Nature.

[11] Niu G, Chen X (2010). Vascular endothelial growth factor as an anti-angiogenic target for cancer therapy. Curr. Drug Targets.

[12] Jászai J, Schmidt MHH (2019). Trends and challenges in tumor anti-angiogenic therapies. Cells.

[13] Vasudev NS, Reynolds AR (2014). Anti-angiogenic therapy for cancer: current progress, unresolved questions and future directions. Angiogenesis.

[14] Haibe Y (2020). Resistance mechanisms to anti-angiogenic therapies in cancer. Front. Oncol..

[15] Jain RK (2014). Antiangiogenesis strategies revisited: from starving tumors to alleviating hypoxia. Cancer Cell.

[16] Yang S, Zhao J, Sun X (2016). Resistance to anti-VEGF therapy in neovascular age-related macular degeneration: a comprehensive review. Drug Des. Dev. Ther..

[17] Rezzola S (2015). Therapeutic potential of anti-angiogenic multitarget *N,O*-sulfated *E. coli* K5 polysaccharide in diabetic retinopathy. Diabetes.

[18] Bunnage ME (2011). Getting pharmaceutical R&D back on target. Nat. Chem. Biol..

[19] Hayes A (2015). Key role of publication of clinical data for target validation. Pharmacol. Res. Perspect..

[20] Hay M, Thomas DW, Craighead JL, Economides C, Rosenthal J (2014). Clinical development success rates for investigational drugs. Nat. Biotechnol..

[21] Dowden H, Munro J (2019). Trends in clinical success rates and therapeutic focus. Nat. Rev. Drug Discov..

[22] Emmerich CH (2021). Improving target assessment in biomedical research: the GOT-IT recommendations. Nat. Rev. Drug Discov..

[23] Chen S (2020). Regulation of SPARC family proteins in disorders of the central nervous system. Brain Res. Bull..

[24] Xiaozhen S (2021). Novel truncating and missense variants in SEMA6B in patients with early-onset epilepsy. Front. Cell Dev. Biol..

[25] Koscielny G (2017). Open Targets: a platform for therapeutic target identification and validation. Nucleic Acids Res..

[26] Qian J (2020). A pan-cancer blueprint of the heterogeneous tumor microenvironment revealed by single-cell profiling. Cell Res..

[27] Pecci A, Ma X, Savoia A, Adelstein RS (2018). MYH9: structure, functions and role of non-muscle myosin IIA in human disease. Gene.

[28] Zhong X, Drgonova J, Li C-Y, Uhl GR (2015). Human cell adhesion molecules: annotated functional subtypes and overrepresentation of addiction-associated genes. Ann. N. Y. Acad. Sci..

[29] Favara DM, Banham AH, Harris AL (2019). ADGRL4/ELTD1 is a highly conserved angiogenesis-associated orphan adhesion GPCR that emerged with the first vertebrates and comprises 3 evolutionary variants. BMC Evol. Biol..

[30] Goodenough DA, Paul DL (2009). Gap junctions. Cold Spring Harb. Perspect. Biol..

[31] Chen HY, Bohlen JF, Maher BJ (2021). Molecular and cellular function of transcription factor 4 in Pitt-Hopkins syndrome. Dev. Neurosci..

[32] Du X, Wang Q, Hirohashi Y, Greene MI (2006). DIPA, which can localize to the centrosome, associates with p78/MCRS1/MSP58 and acts as a repressor of gene transcription. Exp. Mol. Pathol..

[33] Yang B (2020). MYH9 promotes cell metastasis via inducing angiogenesis and epithelial mesenchymal transition in esophageal squamous cell carcinoma. Int. J. Med. Sci..

[34] Lugano R (2018). CD93 promotes β1 integrin activation and fibronectin fibrillogenesis during tumor angiogenesis. J. Clin. Investig..

[35] Okamoto T, Usuda H, Tanaka T, Wada K, Shimaoka M (2019). The functional implications of endothelial gap junctions and cellular mechanics in vascular angiogenesis. Cancers.

[36] Favara DM (2019). ADGRL4/ELTD1 silencing in endothelial cells induces ACLY and SLC25A1 and alters the cellular metabolic profile. Metabolites.

[37] Kanda S, Miyata Y, Kanetake H (2005). T-cell factor-4-dependent up-regulation of fibronectin is involved in fibroblast growth factor-2-induced tube formation by endothelial cells. J. Cell Biochem..

[38] Tanaka A (2010). Inhibition of endothelial cell activation by bHLH protein E2-2 and its impairment of angiogenesis. Blood.

[39] Huang Y (2006). The angiogenic function of nucleolin is mediated by vascular endothelial growth factor and nonmuscle myosin. Blood.

[40] Tosi GM (2020). The binding of CD93 to multimerin-2 promotes choroidal neovascularization. Invest. Ophthalmol. Vis. Sci..

[41] Favara DM (2021). Elevated expression of the adhesion GPCR ADGRL4/ELTD1 promotes endothelial sprouting angiogenesis without activating canonical GPCR signalling. Sci. Rep..

[42] Masiero M (2013). A core human primary tumor angiogenesis signature identifies the endothelial orphan receptor ELTD1 as a key regulator of angiogenesis. Cancer Cell.

[43] Feng Y (2019). CCDC85B promotes non-small cell lung cancer cell proliferation and invasion. Mol. Carcinog..

[44] Li SS (2016). The HSA21 gene EURL/C21ORF91 controls neurogenesis within the cerebral cortex and is implicated in the pathogenesis of Down Syndrome. Sci. Rep..

[45] Iwai A (2008). Coiled-coil domain containing 85B suppresses the β-catenin activity in a p53-dependent manner. Oncogene.

[46] De Bock K (2013). Role of PFKFB3-driven glycolysis in vessel sprouting. Cell.

[47] Mühleder S, Fernández-Chacón M, Garcia-Gonzalez I, Benedito R (2021). Endothelial sprouting, proliferation, or senescence: tipping the balance from physiology to pathology. Cell. Mol. Life Sci..

[48] Yetkin-Arik B (2019). Endothelial tip cells in vitro are less glycolytic and have a more flexible response to metabolic stress than non-tip cells. Sci. Rep..

[49] Zahra FT, Choleva E, Sajib MS, Papadimitriou E, Mikelis CM (2019). In vitro spheroid sprouting assay of angiogenesis. Methods Mol. Biol..

[50] Benn A (2020). BMP-SMAD1/5 signaling regulates retinal vascular development. Biomolecules.

[51] Moya IM (2012). Stalk cell phenotype depends on integration of Notch and Smad1/5 signaling cascades. Dev. Cell.

[52] Kerr G (2015). A small molecule targeting ALK1 prevents Notch cooperativity and inhibits functional angiogenesis. Angiogenesis.

[53] Ambati J, Fowler BJ (2012). Mechanisms of age-related macular degeneration. Neuron.

[54] Yeo NJY, Chan EJJ, Cheung C (2019). Choroidal neovascularization: mechanisms of endothelial dysfunction. Front. Pharmacol..

[55] Carmeliet P, Jain RK (2000). Angiogenesis in cancer and other diseases. Nature.

[56] Lambert V (2013). Laser-induced choroidal neovascularization model to study age-related macular degeneration in mice. Nat. Protoc..

[57] Conchinha NV (2021). Protocols for endothelial cell isolation from mouse tissues: brain, choroid, lung, and muscle. STAR Protoc..

[58] Niinivirta M (2020). Tumor endothelial ELTD1 as a predictive marker for treatment of renal cancer patients with sunitinib. BMC Cancer.

[59] Teixeira JR, Szeto RA, Carvalho VMA, Muotri AR, Papes F (2021). Transcription factor 4 and its association with psychiatric disorders. Transl. Psychiatry.

[60] Heskes T, Eisinga R, Breitling R (2014). A fast algorithm for determining bounds and accurate approximate *p*-values of the rank product statistic for replicate experiments. BMC Bioinformatics.

[61] Hong F (2006). RankProd: a bioconductor package for detecting differentially expressed genes in meta-analysis. Bioinformatics.

[62] Storey JD, Tibshirani R (2003). Statistical significance for genomewide studies. Proc. Natl Acad. Sci. USA.

[63] Taverna F (2020). BIOMEX: an interactive workflow for (single cell) omics data interpretation and visualization. Nucleic Acids Res..

[64] Kalucka J (2020). Single-cell transcriptome atlas of murine endothelial cells. Cell.

[65] Stuart T (2019). Comprehensive integration of single-cell data. Cell.

[66] Schoors S (2015). Fatty acid carbon is essential for dNTP synthesis in endothelial cells. Nature.

[67] Schoors S (2014). Partial and transient reduction of glycolysis by PFKFB3 blockade reduces pathological angiogenesis. Cell Metab..

[68] Guzman-Aranguez A, Loma P, Pintor J (2013). Small-interfering RNAs (siRNAs) as a promising tool for ocular therapy. Br. J. Pharmacol..

[69] Rohlenova K (2020). Single-cell RNA sequencing maps endothelial metabolic plasticity in pathological angiogenesis. Cell Metab..

[70] Voigt AP (2019). Single-cell transcriptomics of the human retinal pigment epithelium and choroid in health and macular degeneration. Proc. Natl Acad. Sci. USA.

[71] Lehmann GL (2020). Single-cell profiling reveals an endothelium-mediated immunomodulatory pathway in the eye choroid. J. Exp. Med..

