# Supplementary material for: Prioritization and functional validation of target genes from single-cell transcriptomics studies
Source: Commun Biol. 2023 Jun 17;6:648. doi: 10.1038/s42003-023-05006-7 (PMC10276815; doi:10.1038/s42003-023-05006-7)
Supplement: Supplementary file 1 — Supplementary Information [file 42003_2023_5006_MOESM1_ESM.pdf]

SUPPLEMENTARY INFORMATION

- SUPPLEMENTARY FIGURES
- SUPPLEMENTARY TABLE 1
- SUPPLEMENTARY REFERENCES

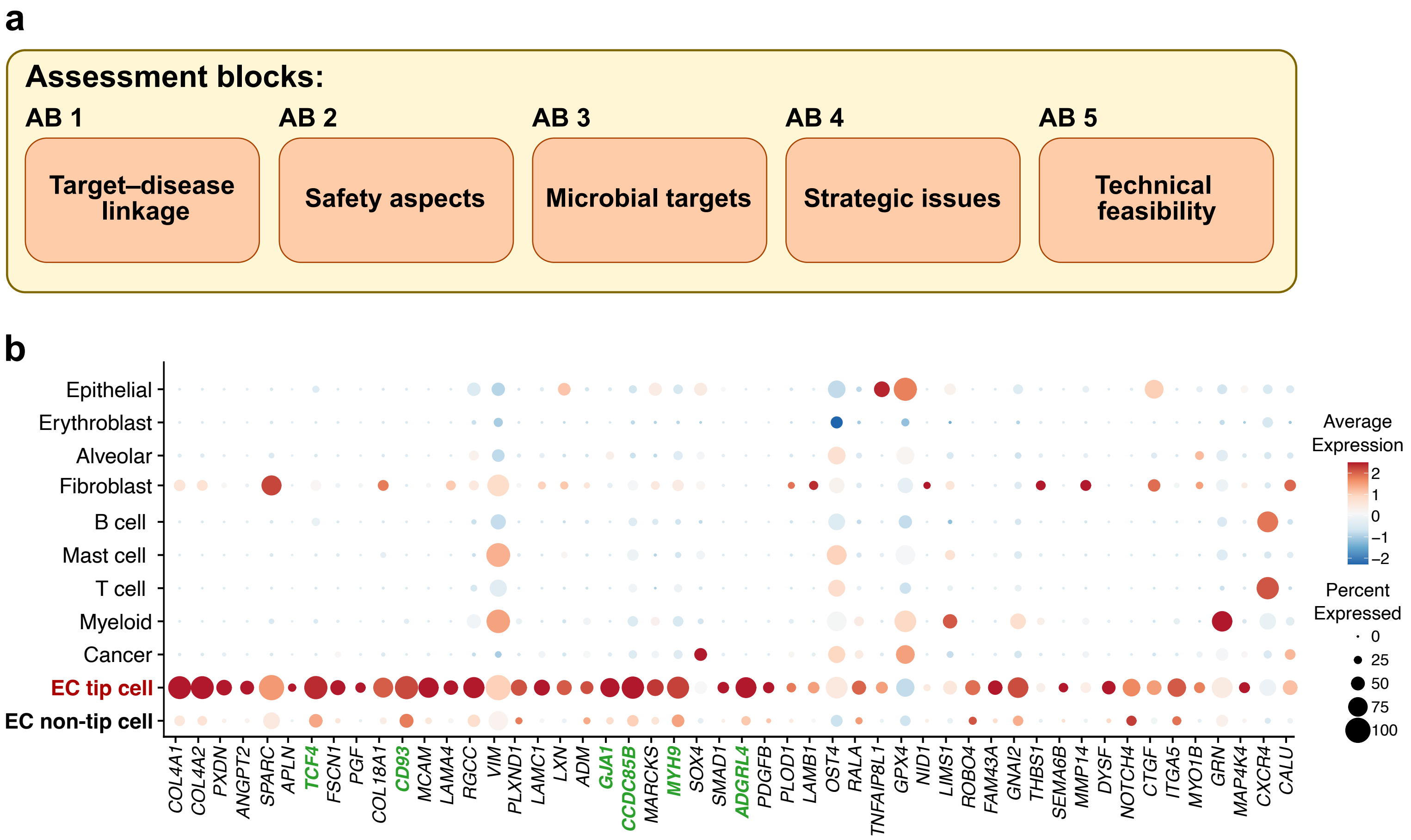
a
Assessment blocks:
AB 1
Target–disease linkage
AB 2
Safety aspects
AB 3
Microbial targets
AB 4
Strategic issues
AB 5
Technical feasibility
b
Epithelial
Erythroblast
Alveolar
Fibroblast
B cell
Mast cell
T cell
Myeloid
Cancer
EC tip cell
EC non-tip cell
COL4A1
COL4A2
PXDN
ANGPT2
SPARC
APLN
TCF4
FSCN1
PGF
COL18A1
CD93
MCAM
LAMA4
RGCC
VIM
PLXND1
LAMC1
LXN
ADM
GJA1
CCDC85B
MARCKS
MYH9
SOX4
SMAD1
ADGRL4
PDGFB
PLOD1
LAMB1
OST4
RALA
TNFAIP8L1
GPX4
NID1
LIMS1
ROBO4
FAM43A
GNAI2
THBS1
SEMA6B
MMP14
DYSF
NOTCH4
CTGF
ITGA5
MYO1B
GRN
MAP4K4
CXCR4
CALU
Average Expression
2
1
0
−1
−2
Percent Expressed
0
25
50
75
100

Supplementary Figure 1: Selection strategy

(a) Schematic representation of Assessment blocks (AB), used for target gene selection/prioritization, adapted from Emmerich *et al.*[1]. **(b)** Dot plot heatmap of the expression of top-50 tip cell markers[2] across cell types in the lung tumor microenvironment[3]. The color intensity of each dot represents the average level of marker gene expression, while the dot size reflects the percentage of cells expressing the marker within the cell subcluster. Color scale: red – high expression, blue – low expression. The green font color marks the genes selected for functional validation. EC – endothelial cell.

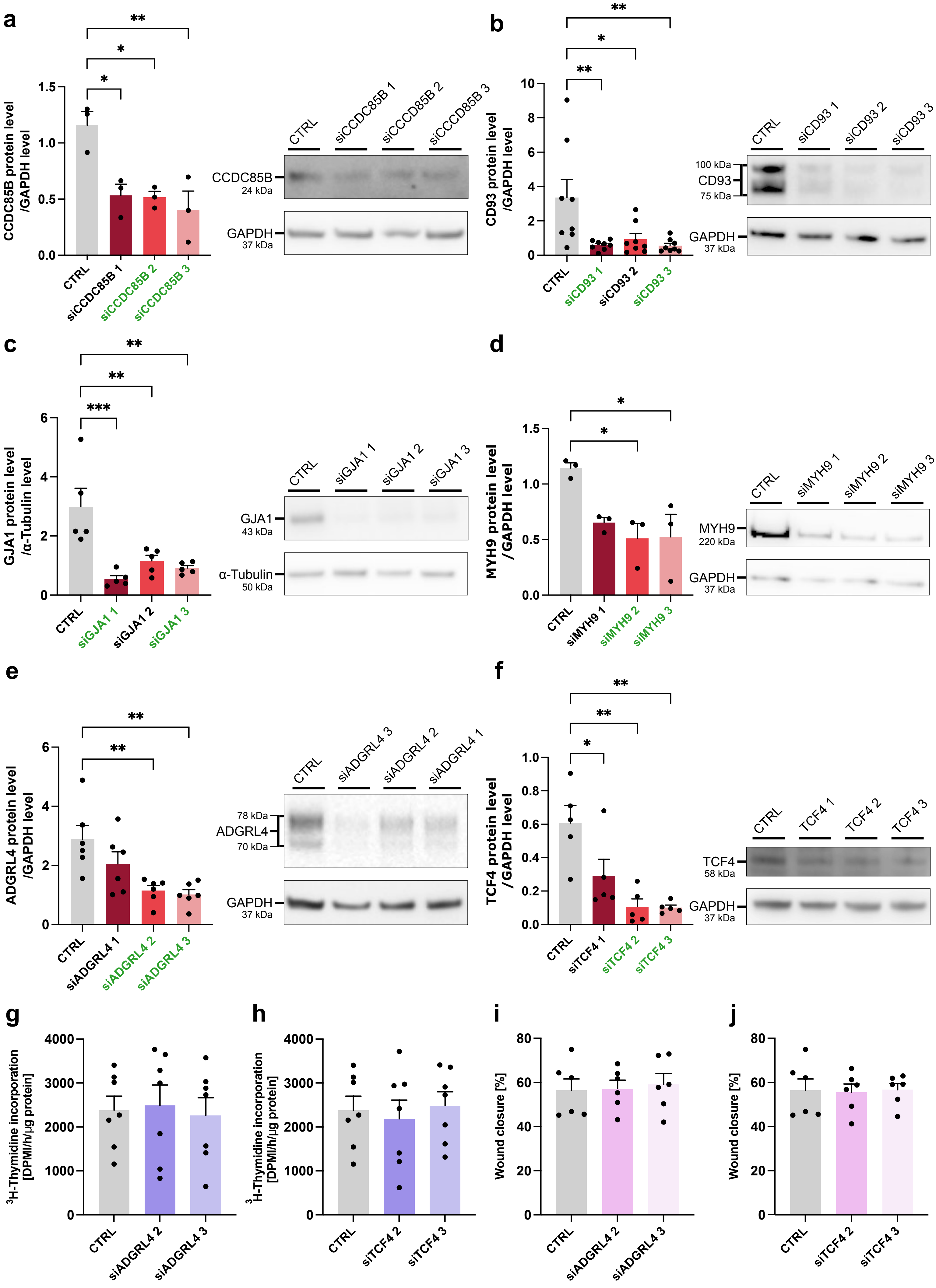

a
CCDC85B protein level /GAPDH level
CTRL
siCCDC85B 1
siCCDC85B 2
siCCDC85B 3
CCDC85B
24 kDa
GAPDH
37 kDa
b
CD93 protein level /GAPDH level
CTRL
siCD93 1
siCD93 2
siCD93 3
100 kDa
CD93
75 kDa
GAPDH
37 kDa
c
GJA1 protein level /α-Tubulin level
CTRL
siGJA1 1
siGJA1 2
siGJA1 3
GJA1
43 kDa
α-Tubulin
50 kDa
d
MYH9 protein level /GAPDH level
CTRL
siMYH9 1
siMYH9 2
siMYH9 3
MYH9
220 kDa
GAPDH
37 kDa
e
ADGRL4 protein level /GAPDH level
CTRL
siADGRL4 1
siADGRL4 2
siADGRL4 3
78 kDa
ADGRL4
70 kDa
GAPDH
37 kDa
f
TCF4 protein level /GAPDH level
CTRL
siTCF4 1
siTCF4 2
siTCF4 3
TCF4 1
TCF4 2
TCF4 3
TCF4
58 kDa
GAPDH
37 kDa
g
³H-Thymidine incorporation [DPM/h/μg protein]
CTRL
siADGRL4 2
siADGRL4 3
h
³H-Thymidine incorporation [DPM/h/μg protein]
CTRL
siTCF4 2
siTCF4 3
i
Wound closure [%]
CTRL
siADGRL4 2
siADGRL4 3
j
Wound closure [%]
CTRL
siTCF4 2
siTCF4 3

Supplementary Figure 2: Silencing effect, proliferation & migration of *ADGRL4* and *TCF4*

(a-f) Representative immunoblot (right) and densitometric quantification (left) of protein level in control and *CCDC85B* (a), *CD93* (b), *GJA1* (c), *MYH9* (d), *ADGRL4* (e), or *TCF4* (f) silenced HUVECs. GAPDH or α-tubulin were used as a loading control. Data are means ± SEM; n=3 (a, d), n=8 (b), n=5 (c, f), n=6 (e); *p < 0.05, **p < 0.01, ***p < 0.001; by one-way ANOVA with Tukey post-hoc test for multiple group comparisons. The green font color marks the siRNA with the strongest knockdown efficiency pre-selected for further *in vitro* validation. Exact p values: siCCDC85B: 1 – 0.0236, 2 – 0.0205, 3 – 0.0086; siCD93 1 – 0.009, 2 – 0.0252, 3 – 0.0081; siGJA1 1 – 0.0006, 2 – 0.0074, 3 – 0.0026; siMYH9 1 – 0.0958, 2 – 0.0318, 3 – 0.035; siADGRL4 1 – 0.3106, 2 – 0.0069, 3 – 0.0037; siTCF4 1 - 0.0435, 2 – 0.0014, 3 – 0.0013. **(g,h)** Quantification of proliferation measured by [^{3}H]-thymidine incorporation in control and *ADGRL4* (g), or *TCF4* (h) silenced HUVECs. Data are means ± SEM; n=7; not significant by one-way ANOVA with Tukey post-hoc test for multiple group comparisons. The assays for *ADGLRL4* and *TCF4* were performed in the same experiment and have the same control values in the quantifications. Exact p values: siADGRL4 2 – 0.9806, 3 – 0.976; siTCF4 2 – 0.9188, 3 – 0.9779. **(i, j)** Quantification of migration by scratch wound assay using control and *ADGLRL4* (i), and *TCF4* (j) silenced HUVECs. MitomycinC-treated HUVECs were used to eliminate confounding effects of proliferation. Data are means ± SEM; n=6; not significant by one-way ANOVA with Tukey post-hoc test for multiple group comparisons. The assays for *ADGLRL4* and *TCF4* were performed in the same experiment and have the same control values in the quantifications. Exact p values: siADGRL4 2 – 0.9928, 3 – 0.9188; siTCF4 2 – 0.9867, 3 – 0.9994. CTRL – HUVECs transfected with control siRNA.

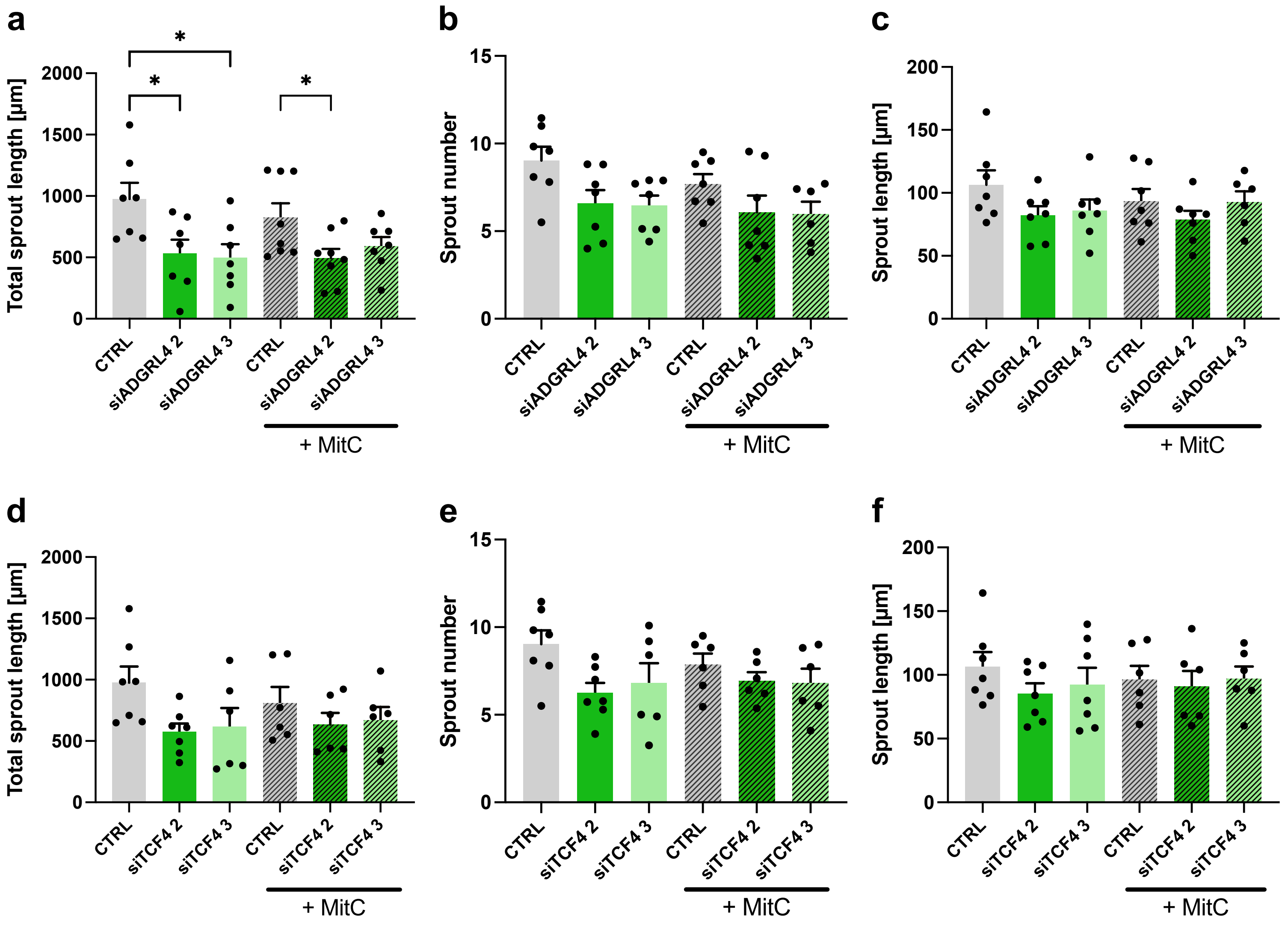

a
b
c
d
e
f
Total sprout length [µm]
Sprout number
Sprout length [µm]
CTRL
siADGRL4 2
siADGRL4 3
siTCF4 2
siTCF4 3
+ MitC
*

SUPPLEMENTARY FIGURE 3: SPROUTING OF *ADGRL4* AND *TCF4*

(a-f) Morphometric quantification of spheroid sprouting using control and *ADGRL4* silenced (a-c) or *TCF4* silenced (d-f) HUVECs with and without Mitomycin C (MitC). For both genes, three sprouting parameters were quantified: cumulative sprout length (a, d), sprout number (b, e), and average sprout length. (c, f). The assays for *ADGRL4* and *TCF4* for n=6 were performed in the same experiment and have the same control values in the quantifications. The remaining replicate for *ADGRL4* + MitC was assayed separately. Data are means ± SEM; n=6-7; *p < 0.05; by one-way ANOVA with Tukey post-hoc test for multiple group comparisons. Exact p values: siADGRL4: total sprout length: 2 – 0.0422, 3 – 0.0276, 2 (+ MitC) – 0.0434, 3 (+ MitC) – 0.2011; sprout number: 2 – 0.0636, 3 – 0.0506, 2 (+ MitC) – 0.3027, 3 (+ MitC) – 0.2915; siTCF4: total sprout length: 2 – 0.0655, 3 – 0.1226, 2 (+ MitC) – 0.5237, 3 (+ MitC) 0.6628; sprout number: 2 – 0.0641, 3 – 0.1776, 2 (+ MitC) – 0.599, 3 (+ MitC) – 0.5244; sprout length: 2 – 0.3854, 3 – 0.6513, 2 (+ MitC) – 0.937, 3 (+ MitC) – 0.9984. CTRL – HUVECs transfected with control siRNA.

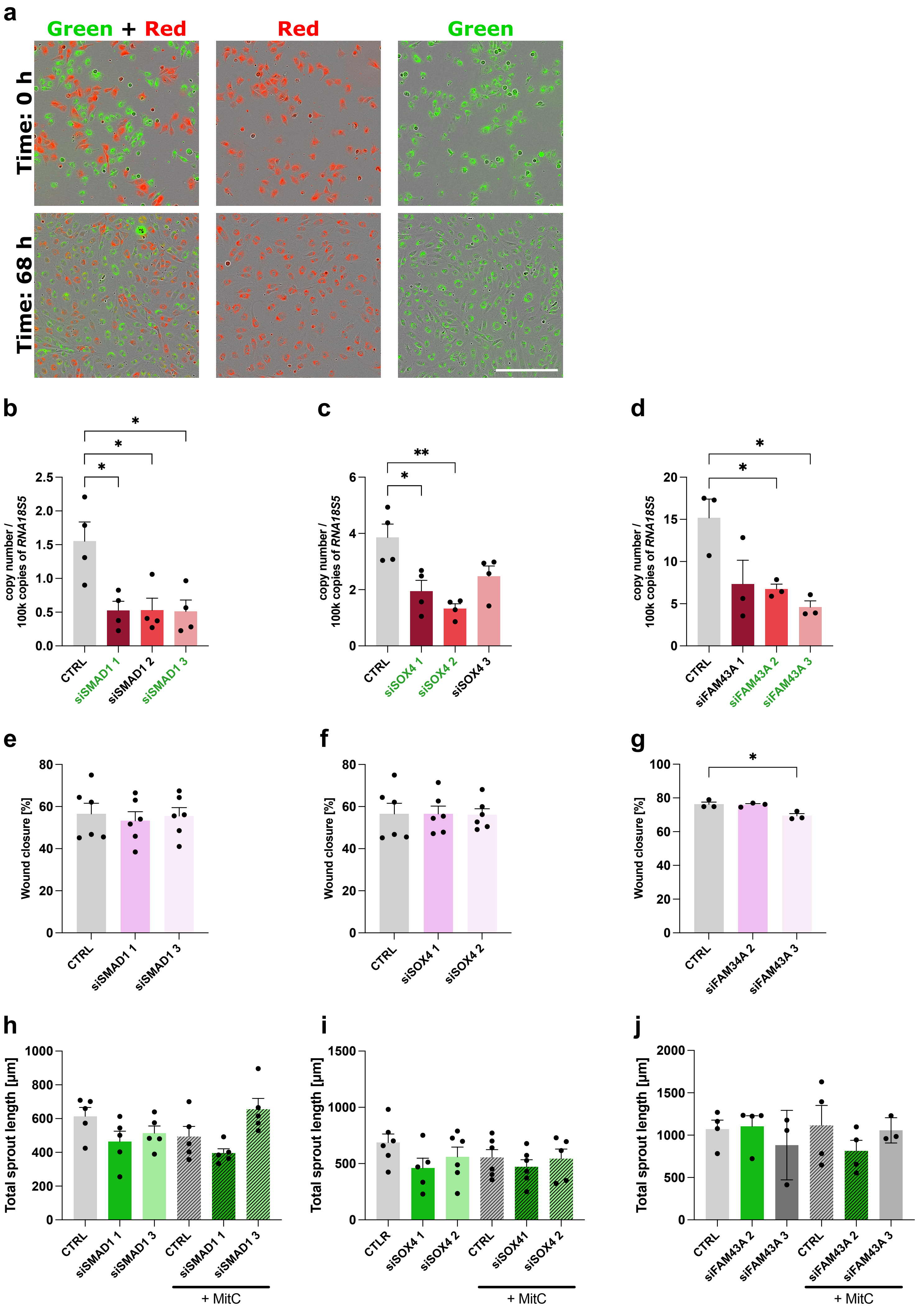

a
Green + Red
Red
Green
Time: 0 h
Time: 68 h
b
copy number / 100k copies of RNA18S5
CTRL
siSMAD1 1
siSMAD1 2
siSMAD1 3
c
CTRL
siSOX4 1
siSOX4 2
siSOX4 3
d
CTRL
siFAM43A 1
siFAM43A 2
siFAM43A 3
e
Wound closure [%]
CTRL
siSMAD1 1
siSMAD1 3
f
CTRL
siSOX4 1
siSOX4 2
g
CTRL
siFAM34A 2
siFAM43A 3
h
Total sprout length [µm]
CTRL
siSMAD1 1
siSMAD1 3
+ MitC
i
CTLR
siSOX4 1
siSOX4 2
CTRL
siSOX41
siSOX4 2
+ MitC
j
CTRL
siFAM43A 2
siFAM43A 3
+ MitC

SUPPLEMENTARY FIGURE 4: TIP CELL COMPETITION AND RANDOM TIP CELL MARKER VALIDATION *IN VITRO*.

(a) Representative fluorescence micrographs showing stability of staining intensity and staining pattern for the entire duration of the *in vitro* tip cell competition assay. HUVECs were stained with the CYTO-ID® Green and/or CYTO-ID® Red long-term cell tracer kit (Enzo) with the same protocol as for tip cell competition assays. Representative fluorescence micrographs show HUVEC cultures immediately after staining (upper panels) and at 68 hours after staining – the endpoint of the tip cell competition assay (lower panels). Left: 1:1 CYTO-ID® Green and CYTO-ID® Red; middle: CYTO-ID® Red; right: CYTO-ID® Green. Scale bar: 300 μm. **(b-d)** mRNA expression levels of *SMAD1* (b), *SOX4* (c) and *FAM43A* (d) in HUVECs in control conditions and upon silencing with 3 independent siRNAs each, measured by RT-qPCR. Data are means ± SEM; n=4 (b, c) or n=3 (d); *p < 0.05, **p < 0.01; by one-way ANOVA with Tukey post-hoc test for multiple group comparisons. The green font color marks the siRNAs with the strongest knockdown efficiency pre-selected for further in vitro validation. Exact p values: siSMAD1: 1 – 0.0159, 2 – 0.0162, 3 – 0.0145; siSOX4: 1 – 0.0141, 2 – 0.0018, 3 – 0.0836; siFMA43A: 1 – 0.0687, 2 – 0.0495, 3 – 0.0162. **(e-g)** Quantification of migration by scratch wound assay using control and *SMAD1* (e), *SOX4* (f) and *FAM43A* (g) silenced HUVECs. Mitomycin C-treated HUVECs were used to eliminate confounding effects of proliferation. Data are means ± SEM; n=6 (*SMAD1* and *SOX4*), n=3 (*FAM43A*); *p < 0.05; by one-way ANOVA with Tukey post-hoc test for multiple group comparisons. The assays for *SMAD1* and *SOX4* were performed in the same experiment and have the same control values in the quantifications. Exact p values: siSMAD1: 1 – 0.8689, 3 – 0.9871; siSOX4: 1 – >0.9999, 2 – 0.9976; siFAM43A: 2 – 0.9827, 3 – 0.0157. **(h-j)** Morphometric quantification of cumulative sprout length of spheroid sprouting using control and *SMAD1* (h), *SOX4* (i) and *FAM43A* (j) silenced HUVECs with and without Mitomycin C (MitC). Data are means ± SEM; n=5 (*SMAD1*), n=6 (*SOX4*), n=4 (siFAM43A 2), n=3 (siFAM43A 3); not significant by one-way ANOVA with Tukey post-hoc test for multiple group comparisons. Exact p values: siSMAD1: 1 – 0.2275, 3 – 0.5546, 1 (+ MitC) – 0.6336, 3 (+ MitC) – 0.2289 ; siSOX4: 1 – 0.2062, 2 – 0.6323, 1 (+ MitC) – 0.8536, 2 (+ MitC) – 0.9995; siFAM43A: 2 – 0.9887, 3 – 0.6737, 2 (+ MitC) – 0.4512, 3 (+ MitC) – 0.9738. CTRL – HUVECs transfected with control siRNA.

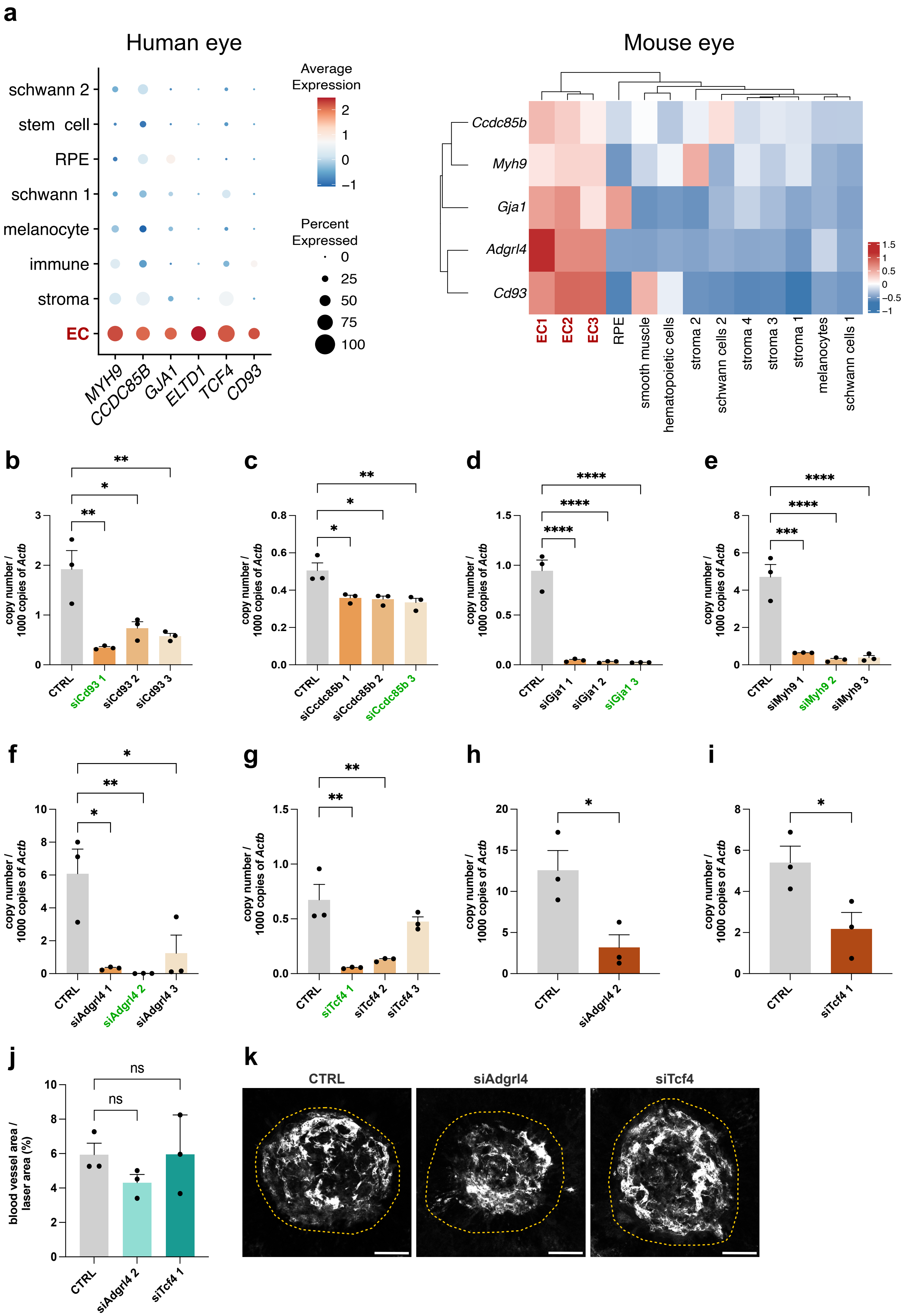

a
Human eye
Mouse eye
schwann 2
stem cell
RPE
schwann 1
melanocyte
immune
stroma
EC
MYH9
CCDC85B
GJA1
ELTD1
TCF4
CD93
Average Expression
Percent Expressed
Ccdc85b
Myh9
Gja1
Adgrl4
Cd93
EC1
EC2
EC3
RPE
smooth muscle
hematopoietic cells
stroma 2
schwann cells 2
stroma 4
stroma 3
stroma 1
melanocytes
schwann cells 1
b
c
d
e
f
g
h
i
j
k
copy number / 1000 copies of Actb
CTRL
siCd93 1
siCd93 2
siCd93 3
siCcdc85b 1
siCcdc85b 2
siCcdc85b 3
siGja1 1
siGja1 2
siGja1 3
siMyh9 1
siMyh9 2
siMyh9 3
siAdgrl4 1
siAdgrl4 2
siAdgrl4 3
siTcf4 1
siTcf4 2
siTcf4 3
blood vessel area / laser area (%)
ns
siAdgrl4
siTcf4

Supplementary Figure 5: *In vivo* model optimization.

(a) (left panel) Dot plot heatmap of the expression of selected tip cell markers across cell types in human retinal pigment epithelium and choroid tissue. The color intensity of each dot represents the average level of marker gene expression, while the dot size reflects the percentage of cells expressing the marker within the cell subcluster. (right panel) Hierarchical clustering heatmap visualization of the expression of selected tip cell markers across cell types in mouse retinal pigment epithelium and choroid tissue. Expression is represented as the log-fold change in expression of a gene in the cell type of interest, compared to all other cell types present in the dataset. Color scale: red – high log-fold change, blue – low log-fold change; data: human: Voigt et al., 2019[4], murine: Lehmann et al., 2020[5]. RPE = retinal pigment epithelium. Note: Tcf4 was not detected in the mouse eye data; multiple clusters of the same cellular subtype are indicated with numbers (*e.g.*, EC1, EC2, etc.). **(b-g)** Expression levels of *Cd93* (b), *Ccdc85b* (c), *Gja1* (d), *Myh9* (e), *Adgrl4* (f), and *Tcf4* (g) *in vitro* in bEnd3 cells in control conditions and upon silencing with 3 independent siRNAs each, measured by RT-qPCR. Data are means ± SEM; n=3; *p < 0.05, **p < 0.01, ***p < 0.001, ****p < 0.0001; by one-way ANOVA with Tukey post-hoc test for multiple group comparisons. CTRL – bEnd3 cells transfected with control siRNA. The green font color marks the siRNA with the strongest knockdown efficiency pre-selected for further *in vivo* validation. Exact p values: siCd93: 1 – 0.0024, 2 – 0.0133, 3 – 0.0064; siCcdc85b 1 – 0.0177, 2 – 0.0139, 3 – 0.0075; siGja1 1-3 < 0.0001; siMyh9 1 – 0.0001, 2, 3 < 0.0001; siAdgrl4 1 – 0.0102, 2 – 0.0075, 3 – 0.0259; siTcf4 1 – 0.0017, 2 – 0.0038, 3 – 0.3048. **(h, i)** Expression levels of *Adgrl4* (h), and *Tcf4* (i), as measured by RT-qPCR in isolated murine choroidal ECs upon siRNA mediated knockdown *in vivo*. Data are means ± SEM; n=3; *p < 0.05; by unpaired two-tailed t-test. Exact p values: siAdgrl4 2 – 0.0314; siTcf4 1 – 0.0469. **(j)** Quantification of CNV blood vessel area in mice treated with control siRNA (CTRL) or siRNA against murine *Adgrl4* or *Tcf4*. Data are means ± SEM; n=3; not significant by one-way ANOVA with Tukey post-hoc test for multiple group comparisons. Each independent experiment was performed using three mice (six eyes per group). Exact p values: siAdgrl4 2 – 0.4549; siTcf4 1 – 09997. **(k)** Representative images of the neovascular area in FITC-dextran (pseudocolored white)-perfused choroidal flat mounts from mice subjected to the CNV model and treated with control siRNA (CTRL) or siRNA against murine *Adgrl4*n or *Tcf4*. The laser area is marked with a yellow dashed line. Scale bar: 75 μm. CTRL – bEnd3 cells (b-g) or mice *in vivo* injected with control siRNA (h-k).

a

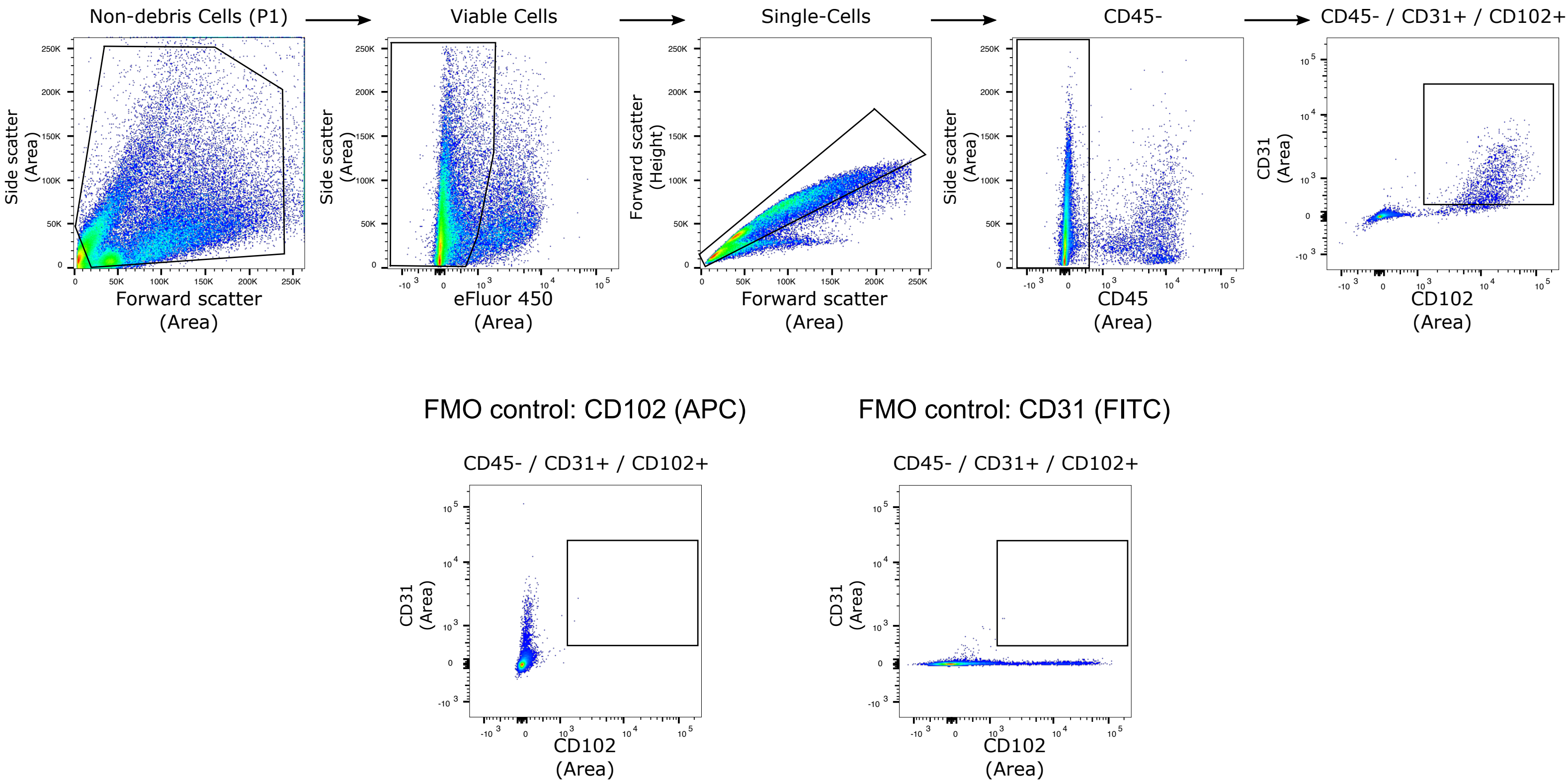
Non-debris Cells (P1)
Viable Cells
Single-Cells
CD45-
CD45- / CD31+ / CD102+
Side scatter (Area)
Forward scatter (Area)
eFluor 450 (Area)
Forward scatter (Height)
CD45 (Area)
CD31 (Area)
CD102 (Area)
FMO control: CD102 (APC)
FMO control: CD31 (FITC)
CD45- / CD31+ / CD102+
CD45- / CD31+ / CD102+

Supplementary Figure 6: Endothelial cell isolation from *in vivo* CNV model.

(a) (top panel) Representative FACS dot plots showing the gating strategy for sorting of ECs from murine choroids based on sorting live/single-cell/$CD45^-$/$CD31^+$/$CD102^+$ cells. Fraction in % of total recorded events: Non-debris Cells (P1): 52.2%; Viable Cells: 45.5%; Single-Cells: 40.7%; CD45-: 37.4%; $CD45^-$/$CD31^+$/$CD102^+$: 1.53%. (bottom panel) Representative FACS dot plots showing the CD45-/$CD31^+$/$CD102^+$ gate in the FMO (Fluorescence Minus One) control sample for CD102 (APC; left) and CD31 (FITC; right).

a

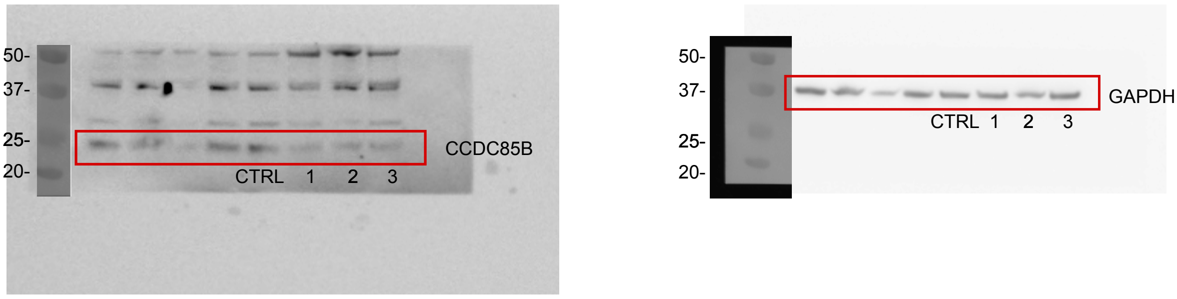
50-
37-
25-
20-
CCDC85B
CTRL 1 2 3
50-
37-
25-
20-
GAPDH
CTRL 1 2 3

b

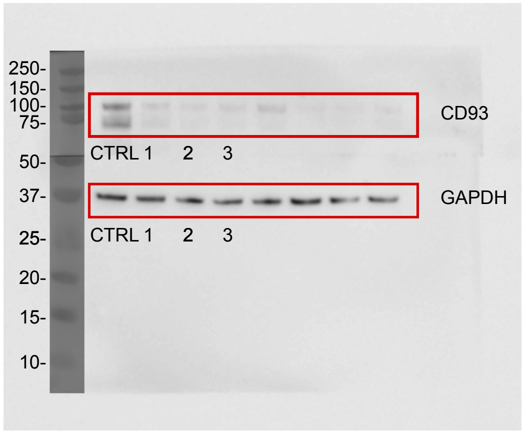
250-
150-
100-
75-
50-
37-
25-
20-
15-
10-
CD93
CTRL 1 2 3
GAPDH
CTRL 1 2 3

c

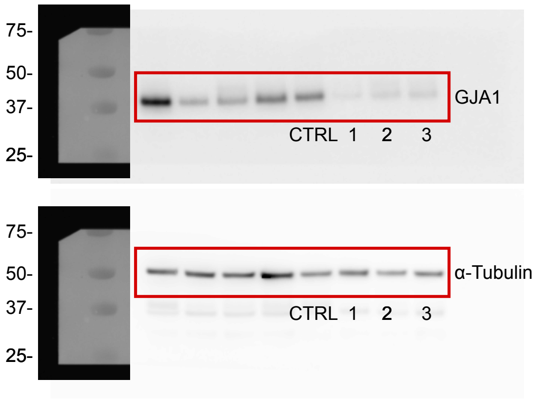
75-
50-
37-
25-
GJA1
CTRL 1 2 3
75-
50-
37-
25-
α-Tubulin
CTRL 1 2 3

d

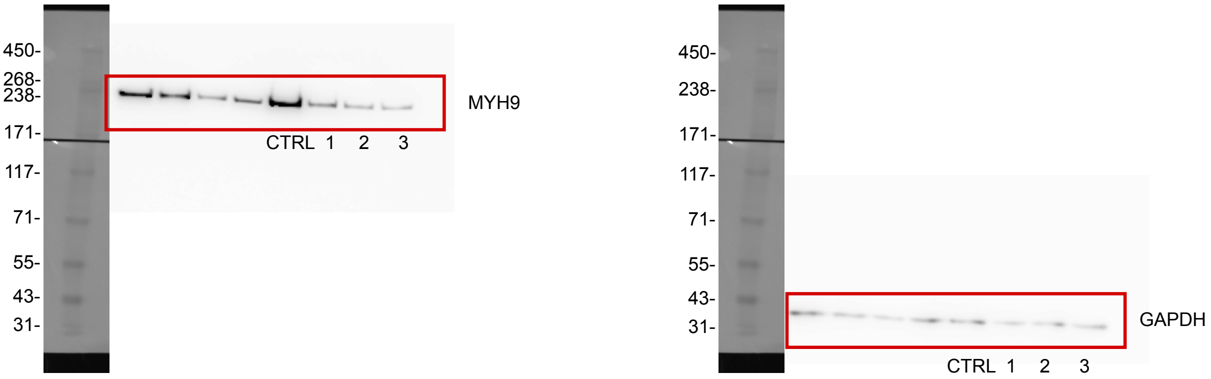
450-
268-
238-
171-
117-
71-
55-
43-
31-
MYH9
CTRL 1 2 3
450-
238-
171-
117-
71-
55-
43-
31-
GAPDH
CTRL 1 2 3

e

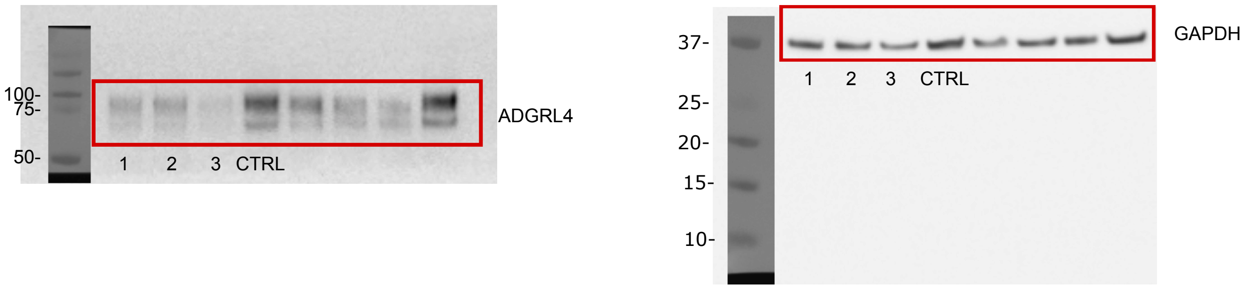
100-
75-
50-
ADGRL4
1 2 3 CTRL
37-
25-
20-
15-
10-
GAPDH
1 2 3 CTRL

f

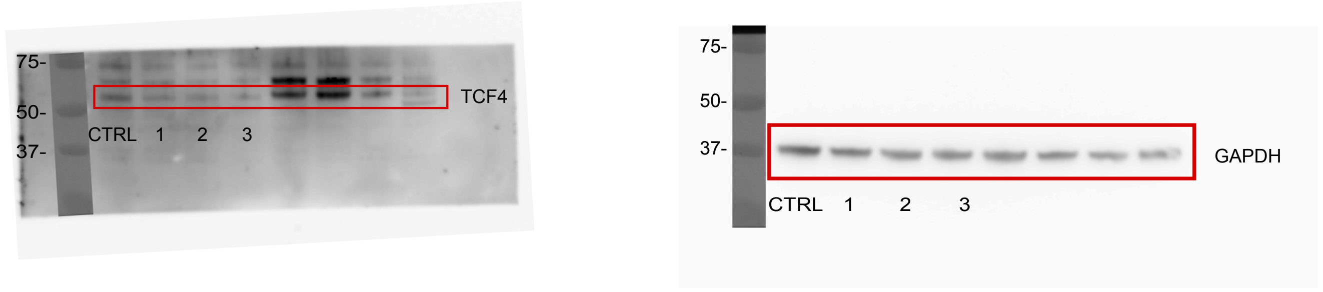
75-
50-
37-
TCF4
CTRL 1 2 3
75-
50-
37-
GAPDH
CTRL 1 2 3

SUPPLEMENTARY FIGURE 7: UNCROPPED AND UNEDITED BLOT IMAGES

Individual panels **(a-f)** show the uncropped blot images with size markers from Supplementary figure 2a-f, respectively. Red boxes indicate the lanes shown in the figures. Depending on the expected band sizes, blots were cut appropriately to allow for immunoblotting of the respective proteins and loading controls.

SUPPLEMENTARY TABLES

SUPPLEMENTARY TABLE 1: RESOURCE TABLE OF REAGENTS, MATERIALS, AND EQUIPMENT

REAGENT or RESOURCE	SOURCE	IDENTIFIER
**Antibodies**		
Anti-α-Tubulin; clone: DM1A (dilution: 1:1000)	Sigma-Aldrich	Cat#T6199
Anti-CCDC85B Antibody; polyclonal (dilution: 1:500)	Proteintech	Cat#18282-1-AP
Anti-CD31 Antibody (FITC); clone: 390 (dilution: 1:100)	Thermo Fisher Scientific	Cat#11-0311-82<br>RRID: AB_465012
Anti-CD45 Antibody (PE-Cy7); clone: 30-F11 (dilution: 1:500)	Thermo Fisher Scientific	Cat#25-0451-82<br>RRID: AB_2734986
Anti-Connexin 43 Antibody (GJA1); clone: F-7 (dilution: 1:500)	Santa Cruz Biotechnology	Cat#SC-271837
Anti-CD93 Antibody; clone: R139 (dilution: 1:500)	Thermo Fisher Scientific	Cat#14-0939-82
Anti-ICAM-2 Antibody (CD102), Alexa Fluor 647; clone: 3C4 (dilution: 1:50)	Thermo Fisher Scientific	Cat#A15452<br>RRID: AB_2534465
Anti-ELTD1 Antibody; clone: CL4164 (dilution: 1:500)	Thermo Fisher Scientific	Cat#MA5-24705
Anti-GAPDH; clone: 14C10 (dilution: 1:1000)	Cell Signaling	Cat#2118
Anti-MYH9 Antibody; polyclonal (dilution: 1:500)	Thermo Fisher Scientific	Cat#PA5-17025
Anti-TCF4 Antibody; clone: D-4 (dilution: 1:500)	Santa Cruz Biotechnology	Cat#SC-166699
**Chemicals, Peptides, and Recombinant Proteins**		
[^{3}H]-thymidine	Perkin Elmer	Cat#NET355L005MC
ACK Lysing Buffer	Gibco	Cat#A10491-01
Antibiotic-antimycotic	Thermo Fisher Scientific	Cat#15240062
Bovine serum albumin (BSA Fraction V)	Sigma-Aldrich	Cat#10735086001
Collagenase type I	Thermo Fisher Scientific	Cat#17018029
Dispase	Thermo Fisher Scientific	Cat#17105-041
DMEM, high glucose	Thermo Fisher Scientific	Cat#11965092
DNase I	Sigma-Aldrich	Cat#D4527-10KU
ECGS (endothelial cell medium growth supplement mix)	PromoCell	Cat#C-39216
EDTA	Sigma-Aldrich	Cat#ED2P-500G
EGM2 (Endothelial growth medium)	PromoCell	Cat#C-22011

Endothelial cell growth factor supplements (ECGS/Heparin)	PromoCell	Cat#C-30120
Fetal bovine serum (FBS)	Thermo Fisher Scientific	Cat#16000044
FITC-dextran	Sigma-Aldrich	Cat#FD2000S
Fixable Viability Dye eFluor™ 780	Thermo Fisher Scientific	Cat#65-0865-14
Gelatin from bovine skin	Sigma-Aldrich	Cat#G9391
Glutamine	Thermo Fisher Scientific	Cat#25030149
Glycerol Merck	Millipore	Cat#1.04091.1000
Hoechst 33258	Sigma-Aldrich	Cat#B2261
Ketamine 100 mg/mL	Eurovet Animal Health B.V.	NIMATEK
KnockOut™ DMEM	Thermo Fisher Scientific	Cat#10829018
Medium 199, HEPES	Thermo Fisher Scientific	Cat#22340020
MEM NEAA	Thermo Fisher Scientific	Cat#11140035
Mitomycin C	Sigma-Aldrich	Cat#M0503-2MG
Methylcellulose 4000 cP	Sigma-Aldrich	Cat#M0512
Opti-MEM	Thermo Fisher Scientific	Cat#11058021
Paraformaldehyde Merck	Millipore	Cat#8.18715.1000
Penicillin/streptomycin	Thermo Fisher Scientific	Cat#15140122
Phosphate buffered saline (DPBS)	Thermo Fisher Scientific	Cat#14190094
RIPA Lysis and Extraction Buffer	Thermo Fisher Scientific	Cat#89901
Phosphatase inhibitors (PhosSTOP)	Roche	Cat# 04906837001
ProLong Gold antifade reagent	Thermo Fisher Scientific	Cat#P36934;
Protease inhibitors (cOmplete, EDTA-free Protease Inhibitor Cocktail)	Roche	Cat#11873580001
Sodium dodecyl sulphate	Acros Organics	Cat#230425000
Sodium pyruvate	Thermo Fisher Scientific	Cat#11360070
SuperSignal West Femto Maximum Sensitivity Substrate	Thermo Fisher Scientific	Cat#34095
TaqMan Fast Universal PCR Master Mix (2X)	Thermo Fisher Scientific	Cat#4364103
TCA (trichloroacetic acid)	Sigma-Aldrich	Cat#T6399
Trypsin-EDTA (0.25%)	Thermo Fisher Scientific	Cat# 25200056
Xylazine	VMD	Cat# XYL-M 2%
**Critical Commercial Assays**		
CYTO-ID® Green long-term cell tracer kit	Enzo	Cat#ENZ-51036-K025
CYTO-ID® Red long-term cell tracer kit	Enzo	Cat#ENZ-51037-K025
in vivo-jetPEI® 0.5 mL	Polyplus	Cat#101000030
Lipofectamine RNAiMAX	Thermo Fisher Scientific	Cat#13778150
Pierce ECL Western Blotting Substrate	Thermo Fisher Scientific	Cat#32106
PureLink RNA Mini Kit	Thermo Fisher Scientific	Cat#12183018A

RNeasy Micro Kit	QIAGEN	Cat#74004
Script cDNA synthesis kit	Bio-Rad	Cat#1708891
SuperScript III First Strand cDNA synthesis kit	Thermo Fisher Scientific	Cat#18080051
**Experimental Models: Organisms/Strains**		
C57BL6/J mice<br>Institutional Animal Ethics Committee of the KU Leuven (Belgium) under protocol numbers P077/2021.	This paper	N/A
Human umbilical vein endothelial cells (HUVECs)<br>Ethics Committee Research KU Leuven/UZ Leuven (approval number S571230)	This paper	N/A
bEnd.3 [BEND3]	ATCC, France	Cat#CRL2299™
**Oligonucleotides**		
Human *CCDC85B* DsiRNA 1	IDT Integrated DNA Technologies	Design ID: hs.Ri.CCDC85B.13.1
Human *CCDC85B* DsiRNA 2	IDT Integrated DNA Technologies	Design ID: hs.Ri.CCDC85B.13.2
Human *CCDC85B* DsiRNA 3	IDT Integrated DNA Technologies	Design ID: hs.Ri.CCDC85B.13.3
Human *CCDC85B* qRT-PCR primer	IDT Integrated DNA Technologies	Cat#NM_006848 Hs.PT.58.3969396.g
Human *CD93* DsiRNA 1	IDT Integrated DNA Technologies	Design ID: hs.Ri.CD93.13.1
Human *CD93* DsiRNA 2	IDT Integrated DNA Technologies	Design ID: hs.Ri.CD93.13.2
Human *CD93* DsiRNA 3	IDT Integrated DNA Technologies	Design ID: hs.Ri.CD93.13.3
Human *CD93* qRT-PCR primer	IDT Integrated DNA Technologies	Cat#NM_012072 Hs.PT.58.45621887.g
Human *ELTD1* DsiRNA 1	IDT Integrated DNA Technologies	Design ID: hs.Ri.ELTD1.13.1
Human *ELTD1* DsiRNA 2	IDT Integrated DNA Technologies	Design ID: hs.Ri.ELTD1.13.2
Human *ELTD1* DsiRNA 3	IDT Integrated DNA Technologies	Design ID: hs.Ri.ELTD1.13.3
Human *ELTD1* qRT-PCR primer	IDT Integrated DNA Technologies	Cat#NM_022159 Hs.PT.58.4652412
Human *FAM43A* qRT-PCR primer	IDT Integrated DNA Technologies	Cat#NM_022159 Hs.PT.58.4652412

Human *FAM43A* DsiRNA 3	IDT Integrated DNA Technologies	Design ID: hs.Ri.ELTD1.13.3
Human *FAM43A* DsiRNA 3	IDT Integrated DNA Technologies	Design ID: hs.Ri.ELTD1.13.3
Human *FAM43A* DsiRNA 3	IDT Integrated DNA Technologies	Design ID: hs.Ri.ELTD1.13.3
Human *GJA1* DsiRNA 1	IDT Integrated DNA Technologies	Design ID: hs.Ri.GJA1.13.1
Human *GJA1* DsiRNA 2	IDT Integrated DNA Technologies	Design ID: hs.Ri.GJA1.13.2
Human *GJA1* DsiRNA 3	IDT Integrated DNA Technologies	Design ID: hs.Ri.GJA1.13.3
Human *GJA1* qRT-PCR primer	IDT Integrated DNA Technologies	Cat#NM_000165 Hs.PT.58.38338544
Human *MYH9* DsiRNA 1	IDT Integrated DNA Technologies	Design ID: hs.Ri.MYH9.13.1
Human *MYH9* DsiRNA 2	IDT Integrated DNA Technologies	Design ID: hs.Ri.MYH9.13.2
Human *MYH9* DsiRNA 3	IDT Integrated DNA Technologies	Design ID: hs.Ri.MYH9.13.3
Human *MYH9* qRT-PCR primer	IDT Integrated DNA Technologies	Cat#NM_002473 Hs.PT.58.26717811
Human *RNA18S5* qRT-PCR primer	IDT Integrated DNA Technologies	Cat#NM_003286 Hs.PT.39a.22214856.g
Human *SMAD1* DsiRNA 1	IDT Integrated DNA Technologies	Design ID: hs.Ri.SMAD1.13.1
Human *SMAD1* DsiRNA 2	IDT Integrated DNA Technologies	Design ID: hs.Ri.SMAD1.13.2
Human *SMAD1* DsiRNA 3	IDT Integrated DNA Technologies	Design ID: hs.Ri.SMAD1.13.3
Human *SMAD1* qRT-PCR primer	IDT Integrated DNA Technologies	Cat# NM_001003688 Hs.PT.58.4596538
Human *SOX4* DsiRNA 1	IDT Integrated DNA Technologies	Design ID: hs.Ri.SOX4.13.1
Human *SOX4* DsiRNA 2	IDT Integrated DNA Technologies	Design ID: hs.Ri.SOX4.13.2
Human *SOX4* DsiRNA 3	IDT Integrated DNA Technologies	Design ID: hs.Ri.SOX4.13.3
Human *SOX4* qRT-PCR primer	IDT Integrated DNA Technologies	Cat# NM_003107 Hs.PT.58.24974948.g

Human *TCF4* DsiRNA 1	IDT Integrated DNA Technologies	Design ID: hs.Ri.TCF4.13.1
Human *TCF4* DsiRNA 2	IDT Integrated DNA Technologies	Design ID: hs.Ri.TCF4.13.2
Human *TCF4* DsiRNA 3	IDT Integrated DNA Technologies	Design ID: hs.Ri.TCF4.13.3
Human *TCF4* qRT-PCR primer	IDT Integrated DNA Technologies	Cat#NM_003199 Hs.PT.58.26293283
Murine *Actb* qRT-PCR prime	IDT Integrated DNA Technologies	Cat#NM_007393 Mm.PT.39a.22214843.g
Murine *Ccdc85b* DsiRNA 1	IDT Integrated DNA Technologies	Design ID: mm.Ri.Ccdc85b.13.1
Murine *Ccdc85b* DsiRNA 2	IDT Integrated DNA Technologies	Design ID: mm.Ri.Ccdc85b.13.2
Murine *Ccdc85b* DsiRNA 3	IDT Integrated DNA Technologies	Design ID: mm.Ri.Ccdc85b.13.3
Murine *Ccdc85b* qRT-PCR primers	IDT Integrated DNA Technologies	Cat#NM_001243307 Mm.PT.58.43843400.g
Murine *Cd93* DsiRNA 1	IDT Integrated DNA Technologies	Design ID: mm.Ri.Cd93.13.1
Murine *Cd93* DsiRNA 2	IDT Integrated DNA Technologies	Design ID: mm.Ri.Cd93.13.2
Murine *Cd93* DsiRNA 3	IDT Integrated DNA Technologies	Design ID: mm.Ri.Cd93.13.3
Murine *Cd93* qRT-PCR prime	IDT Integrated DNA Technologies	Cat#NM_010740 Mm.PT.58.33270099
Murine *Eltd1* DsiRNA 1	IDT Integrated DNA Technologies	Design ID: mm.Ri.ELTD1.13.1
Murine *Eltd1* DsiRNA 2	IDT Integrated DNA Technologies	Design ID: mm.Ri.ELTD1.13.2
Murine *Eltd1* DsiRNA 3	IDT Integrated DNA Technologies	Design ID: mm.Ri.ELTD1.13.3
Murine *Eltd1* qRT-PCR primer	IDT Integrated DNA Technologies	Cat# NM_133222 mm.PT.58.12476951
Murine *Gja1* DsiRNA 1	IDT Integrated DNA Technologies	Design ID: mm.Ri.Gja1.13.1
Murine *Gja1* DsiRNA 2	IDT Integrated DNA Technologies	Design ID: mm.Ri.Gja1.13.2
Murine *Gja1* DsiRNA 3	IDT Integrated DNA Technologies	Design ID: mm.Ri.Gja1.13.3

Murine *Gja1* qRT-PCR primer	IDT Integrated DNA Technologies	Cat#NM_010288 Mm.PT.58.5855325
Murine *Myh9* DsiRNA 1	IDT Integrated DNA Technologies	Design ID: mm.Ri.Myh9.13.1
Murine *Myh9* DsiRNA 2	IDT Integrated DNA Technologies	Design ID: mm.Ri.Myh9.13.2
Murine *Myh9* DsiRNA 3	IDT Integrated DNA Technologies	Design ID: mm.Ri.Myh9.13.3
Murine *Myh9* qRT-PCR prime	IDT Integrated DNA Technologies	Cat#NM_022410 Mm.PT.58.13928218
Murine *Tcf4* DsiRNA 1	IDT Integrated DNA Technologies	Design ID: mm.Ri.TCF4.13.1
Murine *Tcf4* DsiRNA 2	IDT Integrated DNA Technologies	Design ID: mm.Ri.TCF4.13.2
Murine *Tcf4* DsiRNA 3	IDT Integrated DNA Technologies	Design ID: mm.Ri.TCF4.13.3
Murine *Tcf4* qRT-PCR primer	IDT Integrated DNA Technologies	Cat# NM_001083967 mM.PT.58.6779299
Negative Control DsiRNA	IDT Integrated DNA Technologies	Cat#51-01-14-04
**Software and Algorithms**		
BIOMEX	[6]	https://www.vibcancer.be/software-tools/BIOMEX
ComplexHeatmap (version 2.12.1)	[7]	https://www.bioconductor.org/packages/release/bioc/html/ComplexHeatmap.html
Fiji/ImageJ (version 2.3.0 / 1.53f)	https://fiji.sc	RRID: SCR_002285
FlowJo (version 10.8.1)	https://www.flowjo.com	(FlowJo, RRID: SCR_008520)
GraphPad Prism8 (version 9.4)	N/A	(GraphPad Prism, RRID: SCR_002798)
Seurat (v3.1.5)	[8]	https://satijalab.org/seurat/index.html
**Other**		
100 µm cell strainer	Sigma-Aldrich	Cat#CLS431752-50EA
BD FACSAria™ III sorter	BD Biosciences	N/A
Centrifuge tube, conical, HDPE CentriStar™, PP, 15ml	VWR	Cat#734-1867

Centrifuge tube, conical, HDPE CentriStar™, PP, 50ml	VWR	Cat#734-1869
Leica DMI6000 microscope	Leica Microsystems	N/A
Multipurpose- and Micro-centrifuge	N/A	N/A
Nitrocellulose Pre-Cut Blotting Membranes, 0.45 mm pore size	Thermo Fisher Scientific	Cat#LC2001
NuPAGE 10% Bis-Tris Protein Gels, 1.5 mm, 10-well	Thermo Fisher Scientific	Cat#NP0315BOX
NuPAGE 4-12% Bis-Tris Protein Gels, 1.5 mm, 10-well	Thermo Fisher Scientific	Cat#NP0335BOX
NuPAGE LDS Sample Buffer (4X)	Thermo Fisher Scientific	Cat#NP0007
NuPAGE MES SDS Running Buffer (20X)	Thermo Fisher Scientific	Cat#NP0002
NuPAGE MOPS SDS Running Buffer (20X)	Thermo Fisher Scientific	Cat#NP0001
NuPAGE Sample Reducing Agent (10X)	Thermo Fisher Scientific	Cat#NP0009
NuPAGE Transfer Buffer (20X)	Thermo Fisher Scientific	Cat#NP00061
Perfusion pump: Perfusor® fm (MFC)	B. Braun Malaysia	N/A
PUREPOINT®LASER	Alcon	N/A
PVDF Pre-cut Blotting Membranes, 0.2 mm pore size	Thermo Fisher Scientific	Cat#S9549
SOD-peg	Thermo Fisher Scientific	Cat#S9549

Supplementary References

(Supplementary Figures and Supplementary Table 1):

1 Emmerich, C. H. *et al.* Improving target assessment in biomedical research: the GOT-IT recommendations. *Nature Reviews Drug Discovery* **20**, 64-81 (2021). https://doi.org:10.1038/s41573-020-0087-3

2 Goveia, J. *et al.* An Integrated Gene Expression Landscape Profiling Approach to Identify Lung Tumor Endothelial Cell Heterogeneity and Angiogenic Candidates. *Cancer Cell* **37**, 21-36.e13 (2020). https://doi.org:https://doi.org/10.1016/j.ccell.2019.12.001

3 Qian, J. *et al.* A pan-cancer blueprint of the heterogeneous tumor microenvironment revealed by single-cell profiling. *Cell Research* **30**, 745-762 (2020). https://doi.org:10.1038/s41422-020-0355-0

4 Voigt, A. P. *et al.* Single-cell transcriptomics of the human retinal pigment epithelium and choroid in health and macular degeneration. *Proc Natl Acad Sci U S A* **116**, 24100-24107 (2019). https://doi.org:10.1073/pnas.1914143116

5 Lehmann, G. L. *et al.* Single-cell profiling reveals an endothelium-mediated immunomodulatory pathway in the eye choroid. *J Exp Med* **217** (2020). https://doi.org:10.1084/jem.20190730

6 Taverna, F. *et al.* BIOMEX: an interactive workflow for (single cell) omics data interpretation and visualization. *Nucleic Acids Research* **48**, W385-W394 (2020). https://doi.org:10.1093/nar/gkaa332

7 Gu, Z., Eils, R. & Schlesner, M. Complex heatmaps reveal patterns and correlations in multidimensional genomic data. *Bioinformatics* **32**, 2847-2849 (2016). https://doi.org:10.1093/bioinformatics/btw313

8 Stuart, T. *et al.* Comprehensive Integration of Single-Cell Data. *Cell* **177**, 1888-1902.e1821 (2019). https://doi.org:10.1016/j.cell.2019.05.031
