# Supplementary material for: Prioritization and functional validation of target genes from single-cell transcriptomics studies
Source: Commun Biol. 2023 Jun 17;6:648. doi: 10.1038/s42003-023-05006-7 (PMC10276815; doi:10.1038/s42003-023-05006-7)
Supplement: Supplementary file 2 — Description of Additional Supplementary Files [file 42003_2023_5006_MOESM2_ESM.pdf]

Description of Additional Supplementary Files

File name: Supplementary Data 1

Description: Prioritization strategy of tip cell markers as potential candidates for anti-angiogenic therapies and sources used for prioritization strategy of tip cell markers

File name: Supplementary Data 2

Description: Source data of the quantified results of the main figures
