# Supplementary material for: Prioritization and functional validation of target genes from single-cell transcriptomics studies
Source: Commun Biol. 2023 Jun 17;6:648. doi: 10.1038/s42003-023-05006-7 (PMC10276815; doi:10.1038/s42003-023-05006-7)
Supplement: Supplementary file 5 — Reporting Summary [file 42003_2023_5006_MOESM5_ESM.pdf]

Corresponding author(s): Peter Carmeliet

Last updated by author(s): May 16, 2023

Reporting Summary

Nature Portfolio wishes to improve the reproducibility of the work that we publish. This form provides structure for consistency and transparency in reporting. For further information on Nature Portfolio policies, see our Editorial Policies and the Editorial Policy Checklist.

Statistics

For all statistical analyses, confirm that the following items are present in the figure legend, table legend, main text, or Methods section.

n/a	Confirmed	
☐	☒	The exact sample size (*n*) for each experimental group/condition, given as a discrete number and unit of measurement
☐	☒	A statement on whether measurements were taken from distinct samples or whether the same sample was measured repeatedly
☐	☒	The statistical test(s) used AND whether they are one- or two-sided<br>*Only common tests should be described solely by name; describe more complex techniques in the Methods section.*
☒	☐	A description of all covariates tested
☐	☒	A description of any assumptions or corrections, such as tests of normality and adjustment for multiple comparisons
☐	☒	A full description of the statistical parameters including central tendency (e.g. means) or other basic estimates (e.g. regression coefficient) AND variation (e.g. standard deviation) or associated estimates of uncertainty (e.g. confidence intervals)
☐	☒	For null hypothesis testing, the test statistic (e.g. *F*, *t*, *r*) with confidence intervals, effect sizes, degrees of freedom and *P* value noted<br>*Give P values as exact values whenever suitable.*
☒	☐	For Bayesian analysis, information on the choice of priors and Markov chain Monte Carlo settings
☒	☐	For hierarchical and complex designs, identification of the appropriate level for tests and full reporting of outcomes
☒	☐	Estimates of effect sizes (e.g. Cohen's *d*, Pearson's *r*), indicating how they were calculated

Our web collection on statistics for biologists contains articles on many of the points above.

Software and code

Policy information about availability of computer code

Data collection	All datasets analyzed in this study were publicly available, and downloaded from their respective repositories. Human lung cancer EC scRNA-seq data - E-MTAB-6308, and at https://carmelietlab.sites.vib.be/en/software-tools (lung Tumor ECTax); Human lung cancer scRNA-seq data - E-MTAB-8107, and at https://lambrechtslab.sites.vib.be/en/data-access; Human and mouse eye data - GSE135922 (human) and GSE135167 (mouse).
Data analysis	For E-MTAB-8107: The EC subset of the processed data was separately subclustered and annotated using previously reported EC-subtype specific marker genes. Briefly, ECs were selected based on the annotations provided by the study (subset function of Seurat (v3.1.5), idents = 'EC'). Data were normalized using the NormalizeData function, followed by identification of the top-2000 highly variable genes using FindVariableFeatures, and scaling of the data using the ScaleData function. The scaled data was then summarized by principal component analysis (PCA; RunPCA function), and subclustered (FindClusters function, resolution=1), followed by visualization using uniform manifold approximation and projection (UMAP; runUMAP function). EC clusters were annotated based on the expression of known EC and non-EC marker genes, including GJA5 and CXCL12 (arterial ECs), EDNRB, HPGD, TMEM100, BTNL9 (microvascular ECs), ACKR1 and VCAM1 (venous ECs) PROX1 and LYVE1 (lymphatic ECs), APLN and PGF (tip ECs), PTPRC and CD68 (immune cells), DCN, LUM, PDGFRB (stromal cells), EPCAM, CDH1 (epithelial cells). Contaminating immune cell clusters, as well as clusters without any clear EC marker gene expression (but high expression of ribosomal genes and/or a relatively lower number of detected genes) were removed, and all downstream analysis was performed on the finally selected ECs only (n = 3448 cells). EC subclusters were annotated as either tip or non-tip EC, and the ECs were combined again with all non-EC cell types profiled in this dataset (cancer, alveolar, epithelial, myeloid, T, B and mast cells, fibroblasts and erythroblasts). To determine the specificity of our candidate markers to the tip EC cluster, we calculated marker genes for every cell type using the FindAllMarkers function (only.pos=TRUE, max.cells=1000, all other parameters were default). Tip cell markers enriched in the tip EC cluster with a log fold change > 1 (as compared to all other cell types) were considered tip cell and EC specific. After applying all the above-mentioned criteria, 6 potential target genes emerged: CCDC85B, CD93, GJA1, ADGRL4 and TCF4.

For GSE135922: Data were downloaded from the Gene Expression Omnibus (GEO; accession number GSE135922) and analyzed using Seurat (v3.1.5). Briefly, the data was normalized, followed by identification of the top 2000 highly variable genes, and scaling of the data. The resulting data was then summarized by principal component analysis and subclustered (FindClusters function, resolution 0.5, using the top-25 principal components), followed by UMAP visualization. Subclusters were annotated based on cellular lineages and reported marker genes in the original publication, and the DotPlot() function was used for dotplot heatmap visualization of marker genes.
For GSE135167: Data were downloaded from GEO (accession number GSE135167). Log-fold change values were extracted for every candidate gene, followed by heatmap visualization using the ComplexHeatmap R package (2.12.1; Euclidean distance, average linkage). Tcf4 was not present in this dataset.

For manuscripts utilizing custom algorithms or software that are central to the research but not yet described in published literature, software must be made available to editors and reviewers. We strongly encourage code deposition in a community repository (e.g. GitHub). See the Nature Portfolio guidelines for submitting code & software for further information.

Data

Policy information about availability of data

All manuscripts must include a data availability statement. This statement should provide the following information, where applicable:

- Accession codes, unique identifiers, or web links for publicly available datasets
- A description of any restrictions on data availability
- For clinical datasets or third party data, please ensure that the statement adheres to our policy

The publicly available lung cancer EC data used in this study are available in the ArrayExpress database at EMBL-EBI under accession code E-MTAB-6308, and at https://carmelietlab.sites.vib.be/en/software-tools (lung Tumor ECTax). The publicly available lung cancer data used in this study are available in the ArrayExpress database at EMBL-EBI un-der accession code E-MTAB-8107, and at https://lambrechtslab.sites.vib.be/en/data-access. The publicly available human and mouse eye data used in this study are available at the Gene Expression Omnibus (GEO) under accession numbers GSE135922 (human) and GSE135167 (mouse). Source data are provided with this paper.

Human research participants

Policy information about studies involving human research participants and Sex and Gender in Research.

Reporting on sex and gender	HUVECs were obtained from multiple donors of unknown sex
Population characteristics	Except for HUVECs, this study does not involve human participants.
Recruitment	N/A
Ethics oversight	Approval from the Ethics Committee Research KU Leuven / UZ Leuven under the approval number S57123 and informed consent obtained from all subjects.

Note that full information on the approval of the study protocol must also be provided in the manuscript.

Field-specific reporting

Please select the one below that is the best fit for your research. If you are not sure, read the appropriate sections before making your selection.

☒ Life sciences ☐ Behavioural & social sciences ☐ Ecological, evolutionary & environmental sciences

For a reference copy of the document with all sections, see nature.com/documents/nr-reporting-summary-flat.pdf

Life sciences study design

All studies must disclose on these points even when the disclosure is negative.

Sample size	n>3
Data exclusions	N/A
Replication	All reported findings were replicated across multiple independent biological samples (see figure legends for n).
Randomization	No randomization of subjects was performed in this study.
Blinding	N/A

Reporting for specific materials, systems and methods

We require information from authors about some types of materials, experimental systems and methods used in many studies. Here, indicate whether each material, system or method listed is relevant to your study. If you are not sure if a list item applies to your research, read the appropriate section before selecting a response.

Materials & experimental systems

n/a	Involved in the study
☐	☒ Antibodies
☐	☒ Eukaryotic cell lines
☒	☐ Palaeontology and archaeology
☐	☒ Animals and other organisms
☒	☐ Clinical data
☒	☐ Dual use research of concern

Methods

n/a	Involved in the study
☒	☐ ChIP-seq
☐	☒ Flow cytometry
☒	☐ MRI-based neuroimaging

Antibodies

Antibodies used

Anti-α-Tubulin; clone: DM1A (dilution: 1:1000) Sigma-Aldrich Cat#T6199
https://www.sigmaaldrich.com/BE/en/product/sigma/t6199; validated

Anti-CCDC85B Antibody; polyclonal (dilution: 1:500) Proteintech Cat#18282-1-AP
https://www.ptglab.com/products/CCDC85B-Antibody-18282-1-AP.htm; validated

Anti-CD31 Antibody (FITC); clone: 390 (dilution: 1:100) Thermo Fisher Scientific Cat#11-0311-82; RRID: AB_465012
https://www.thermofisher.com/antibody/product/CD31-PECAM-1-Antibody-clone-390-Monoclonal/11-0311-82; validated

Anti-CD45 Antibody (PE-Cy7); clone: 30-F11 (dilution: 1:500) Thermo Fisher Scientific Cat#25-0451-82; RRID: AB_2734986
https://www.thermofisher.com/antibody/product/CD45-Antibody-clone-30-F11-Monoclonal/25-0451-82; validated

Anti-Connexin 43 Antibody (GJA1); clone: F-7 (dilution: 1:500) Santa Cruz Biotechnology Cat#SC-271837
https://www.scbt.com/p/connexin-43-antibody-f-7; validated

Anti-CD93 Antibody; clone: R139 (dilution: 1:500) Thermo Fisher Scientific Cat#14-0939-82
https://www.thermofisher.com/antibody/product/CD93-AA4-1-Antibody-clone-R139-Monoclonal/14-0939-82; validated

Anti-ICAM-2 Antibody (CD102), Alexa Fluor 647; clone: 3C4 (dilution: 1:50) Thermo Fisher Scientific Cat#A15452; RRID: AB_2534465
https://www.thermofisher.com/antibody/product/ICAM-2-Antibody-clone-3C4-Monoclonal/A15452; validated

Anti-ELTD1 Antibody; clone: CL4164 (dilution: 1:500) Thermo Fisher Scientific Cat#MA5-24705
https://www.thermofisher.com/antibody/product/ELTD1-Antibody-clone-CL4164-Monoclonal/MA5-24705; validated

Anti-GAPDH; clone: 14C10 (dilution: 1:1000) Cell Signaling Cat#2118
https://www.cellsignal.com/products/primary-antibodies/gapdh-14c10-rabbit-mab/2118; validated

Anti-MYH9 Antibody; polyclonal (dilution: 1:500) Thermo Fisher Scientific Cat#PA5-17025
https://www.thermofisher.com/antibody/product/MYH9-Antibody-Polyclonal/PA5-17025; validated

Anti-TCF4 Antibody; clone: D-4 (dilution: 1:500) Santa Cruz Biotechnology Cat#SC-166699
https://www.scbt.com/p/tcf-4-antibody-d-4; validated

Validation

All antibodies are commercially available and validated by the respective vendors for the assays and species used in this work.

Eukaryotic cell lines

Policy information about [cell lines and Sex and Gender in Research]

Cell line source(s)	bEnd.3 [BEND3] ATCC, France Cat#CRL2299TM
Authentication	This cell line was not authenticated by us.
Mycoplasma contamination	Negative
Commonly misidentified lines (See ICLAC register)	N/A

Animals and other research organisms

Policy information about studies involving animals; ARRIVE guidelines recommended for reporting animal research, and Sex and Gender in Research

Laboratory animals	C57BL6/J mice
Wild animals	N/A
Reporting on sex	Female and male
Field-collected samples	N/A
Ethics oversight	Institutional Animal Ethics Committee of the KU Leuven (Belgium) under protocol numbers P077/2021.

Note that full information on the approval of the study protocol must also be provided in the manuscript.

Flow Cytometry

Plots

Confirm that:

☒ The axis labels state the marker and fluorochrome used (e.g. CD4-FITC).

☒ The axis scales are clearly visible. Include numbers along axes only for bottom left plot of group (a 'group' is an analysis of identical markers).

☒ All plots are contour plots with outliers or pseudocolor plots.

☒ A numerical value for number of cells or percentage (with statistics) is provided.

Methodology

Sample preparation	Choroidal endothelial cells were isolated based on the protocol published by Conchinha et al., omitting the magnetic cell sorting steps. In brief, on day 7 after the laser-induced CNV (as described above), the mice were sacrificed by cervical dislocation, and eyes were collected by inserting scissors along the eye into the orbital cavity. The four optical muscles and the optic nerve, which appear as a white cord behind the eye, were cut. The dissected eyes were then placed in PBS and periocular tissue was removed. The retinal pigment epithelium (RPE)-choroid-sclera complex was dissected from the enucleated eyes by peeling off the vitreous body and retina. The choroids were dissociated into single-cell suspension in a digestion buffer (0.3% (w/v) collagenase I, DNase I (7.5 ug/mL) and dispase (0.25 U/mL) in Knock-OutTM DMEM-medium (Thermo Fisher Scientific) supplemented with 1mM sodium pyruvate, 1x MEM NEAAs, ECGF/Heparin, antibiotic/antimycotic (2x) and 1% (v/v) penicillin/streptomycin) for 30-40 min at 37oC with manual pipetting every 10 min. The reaction was stopped with 5 mL of wash buff-er57 and the cell suspension was filtered through a 100 and 40 um cell strainer. The choroidal endothelial cells were isolated using Fluorescence-activated Cell Sorting (FACS) (see Supplementary Figure S6a for gating strategy) and subjected to RNA isolation (RNeasy Micro Kit (QIAGEN); SuperScript III First Strand cDNA synthesis kit (Thermo Fisher Scientific)) and quantitative RT-PCR. This procedure was repeated for 4-5 independent replicate experiments, each: three mice (six eyes) per condition.
Instrument	FACS Aria III (BD Biosciences)
Software	FlowJo software (BD Biosciences), version 10.8.1
Cell population abundance	On average, ECs (single, viable, CD45-, CD31+, CD102+) comprised 2-4% of total choroidal cells.
Gating strategy	Single-cell suspensions were stained with viability dye (VD, eFluor™ 450, dilution 1:1000) and fluorescently labeled antibodies (CD45 (PE-Cy7, dilution 1:500), CD31 (AF488, dilution 1:100) and CD102/ICAM2 (APC, dilution 1:50)) for 30 minutes. Then, we FACS-sorted viable single cells (VD–) CD45-, CD102+ CD31+ directly into the lysis buffer from the RNeasy Micro Kit (QIAGEN Cat#74004). We based the selection of ECs both on CD31 and CD102 to increase purity.

☒ Tick this box to confirm that a figure exemplifying the gating strategy is provided in the Supplementary Information.
